# Quantitative Analysis of the Doping and Defect Density in Mixed Sn–Pb Perovskites Mediated by SnF_2_

**DOI:** 10.1021/acs.chemmater.5c00816

**Published:** 2025-10-02

**Authors:** Jasmeen Nespoli, Maartje J. van der Meer, Sander Heester, Jim S. Koning, Bart Boshuizen, L. Jan Anton Koster, Tom J. Savenije

**Affiliations:** † Department of Chemical Engineering, Faculty of Applied Sciences, 2860 Delft University of Technology, Delft 2629 HZ, The Netherlands; ‡ Zernike Institute for Advanced Materials, 3647 University of Groningen, Nijenborgh 3, Groningen 9747AG, The Netherlands

## Abstract

Last year’s mixed Sn–Pb perovskites have been applied as low-bandgap absorbers in efficient solar cells. However, the performance is still limited by tin oxidation, resulting in doping and defects. Here we perform a quantitative analysis on how tin oxidation affects the optoelectronic properties of spin-coated Cs_0.25_FA_0.75_Sn_0.5_Pb_0.5_I_3_ with varying SnF_2_ additions ranging from 0 to 20 mol %. First, optical spectroscopy is used to determine the fraction of Sn^4+^ in the spin-coating solution, which varies depending on the purity of the starting SnI_2_ precursor. By applying steady-state microwave conductance, a large decrease in the dark conductivity from ∼100 to <∼1 S m^–1^ in the spin-coated films on going from 0 to 2 mol % SnF_2_ is observed. We conclude that, without SnF_2_, ∼12% of the Sn^4+^ in solution leads to mobile carriers in the form of free holes, *p*_0_, in the perovskite layer. Upon SnF_2_ addition, *p*_0_ decreases to <1 × 10^16^ cm^–3^. We infer that a ∼70 times excess of SnF_2_ over the initial concentration of Sn^4+^ in solution is required to scavenge the Sn^4+^ and obtain layers with reduced doping. Although the reduction of *p*_0_ and defects results in increased carrier lifetimes, higher SnF_2_ additions are also required to decrease the surface defects, leading to even longer lifetimes close to 200 ns. The reduced doping of these perovskite films with SnF_2_ makes them ideal candidates for efficient solar cells; however, SnF_2_ also induces compositional heterogeneity and accumulation of SnO_*x*_ at the surface.

## Graphical abstract

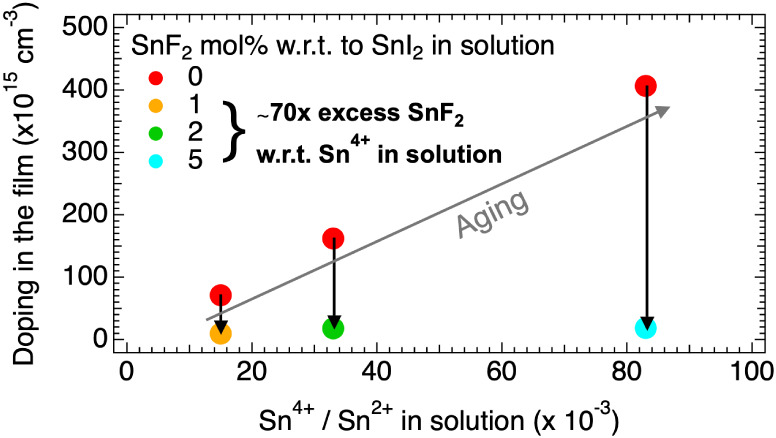

## Introduction

In the past decade, metal halide perovskites (MHPs) have emerged as promising materials for photovoltaics.^1^ Their crystal structure is represented by the formula ABX_3_, where the A-sites can be occupied by an organic or large inorganic cation (methylammonium, MA^+^; formamidinium, FA^+^; cesium, Cs^+^), the B-sites by a divalent metal cation (lead, Pb^2+^; tin, Sn^2+^) and the X-sites by a halide anion (iodide, I^–^; bromide, Br^–^; chloride, Cl^–^).^1^ Apart from research on Pb-based MHPs, mixed Sn–Pb perovskite absorbers have also been applied in low-bandgap single- and multijunction solar cells to attain power conversion efficiencies of around 24% and 28%, respectively.^1–5^ While the incorporation of tin in the perovskite crystal structure could lead to higher efficiencies on the basis of the Shockley–Queisser limit,^1^ the performance of tin-containing perovskites is still substantially below this limit. In the literature, this is often related to the propensity of Sn^2+^ to oxidize to Sn^4+^,^6–10^ leading to doping and/or to the formation of crystal defects such as tin vacancies.^6,11–16^

In Sn-based MHPs, p-type doping is claimed to originate from tin oxidation.^6,10,17,18^ A Sn^4+^ located at a B-site, in the Kröger–Vink notation for crystal defects, $Sn_{Sn}^{\cdot \cdot }$ ,^17^ is claimed to be unstable in the perovskite lattice and may be displaced toward the perovskite surface.^10^ At the same time $Sn_{Sn}^{\cdot \cdot }$ must be compensated by other negatively charged defects to achieve charge neutrality, such as tin vacancies, $V_{\mathrm{Sn}}^{\prime \prime }$ , or iodide interstitials, $2I_{i}^{\prime }$ .^6,10,17^ In Sn-based perovskites, these lattice defects form electron acceptor states below the valence band edge, and consequently, two free holes are generated, leading to p-doping.^6^ However, this explanation is debated for mixed Sn–Pb perovskites, where $V_{\mathrm{Sn}}^{\prime \prime }$ and $I_{i}^{\prime }$ are supposed to form deep traps (surface) and shallow traps (bulk) in the forbidden band.^14,15^ Hence, although a connection between tin oxidation, doping, and crystal defects seems to exist, the underlying mechanism is still not fully clear for mixed Sn–Pb perovskites.

Doping is detrimental for the efficiency of perovskite solar cells (PSCs), as it leads to pseudomonomolecular recombination between photogenerated electrons and the free holes, resulting in short carrier lifetimes.^6,12,13,19^ Besides, in the literature, doping is typically associated with crystal defect formation.^6,8–10,15,20,21^ Although the precise nature of these defects is not yet understood, a high defect density not only enhances nonradiative recombination^6,12,13,19^ but also reduces the carrier mobility through ionized-impurity scattering.^6,13^ A low carrier mobility-lifetime product leads in turn to a short carrier diffusion length.^12,13,19^ All these factors not only affect the photovoltaic performance^20–22^ but also make the crystal more susceptible to degradation.^10^

A plethora of additives has been explored to mitigate these negative effects in mixed Sn–Pb perovskites, with SnF_2_ being particularly popular for solution-based perovskites due to its ability to reduce the concentration of dark free holes in the perovskite layer.^12,13^ It is reported that SnF_2_ can remove oxidized Sn^4+^ by a ligand exchange reaction in the spin-coating solution. Indeed, thanks to the stronger affinity of the small and highly electronegative F^–^ ion to the smaller and more electronegative Sn^4+^ with respect to Sn^2+^, the SnI_4_ in solution can be converted into SnI_2_ and SnF_4_ as shown in eq 1.^23^

1 $$Sn^{\mathrm{IV}}I_{4}+2Sn^{\mathrm{II}}F_{2}\to Sn^{\mathrm{IV}}F_{4}+2Sn^{\mathrm{II}}I_{2}$$

Moreover, it is also reported that SnF_2_ acts as an oxygen scavenger by promoting the formation of tin oxide phases, SnO_2_ or SnO_1.2_F_(0.2–0.5)_, at the film interfaces,^24,25^ and improves both the crystal structure and microstructure of perovskite thin films.^24–26^

To rationalize the effects of tin oxidation in mixed Sn–Pb perovskites and the impact of the SnF_2_ additive, we examined the purity of the SnI_2_ precursor by aging it for different periods. We isolated the resulting oxidation products, i.e., SnI_4_, from the aged SnI_2_ precursors via extraction by toluene and studied the resulting toluene solutions by absorption spectroscopy. Next, perovskite precursor solutions were prepared by using differently aged SnI_2_ and Cs_0.25_FA_0.75_Sn_0.5_Pb_0.5_I_3_ thin films that were deposited. To study how the amount of Sn^4+^ affects the conductivity of the perovskite layers, we added different concentrations of SnF_2_ to the precursor solution, varying from 0 to 20 mol %. By microwave conductance measurements, we quantified the dark conductivity (doping) in the perovskite films. We also examined the photoinduced charge carrier dynamics by time-resolved microwave conductivity (TRMC) and fitted the intensity-dependent photoconductivity TRMC signals with a 1D drift/diffusion model. This enabled us to extract the doping and defect density of the layers, distinguishing between bulk (shallow) and surface (deep) defect states. To couple the absorption spectroscopy and microwave conductivity results, we used the same SnI_2_ precursor and analyzed quantitatively the Sn^4+^ concentration in solution and the doping in the corresponding perovskite layer. In this way, we studied the relationship between the initial level of oxidation of SnI_2_ in solution and the doping and crystal defect densities of the films. Additionally, structural, optical, and elemental composition analyses were performed to clarify the mechanisms governing the optoelectronic properties of these perovskite layers.

## Experimental Section

### Materials

Cesium iodide (CsI, 99.999%) and tin­(II) fluoride (SnF_2_, 99%) were purchased from Merck-Sigma-Aldrich. The organic halide salt formamidinium iodide (FAI, 99.99%) was purchased from Greatcell Solar Materials. Lead­(II) iodide (PbI_2_, 99%) was purchased from Acros Organics. Tin­(II) iodide (SnI_2_, 99.999%, −10 mesh beads) and tin­(IV) fluoride (SnF_4_, 99%, −6 mesh crystalline) were purchased from Alfa Aesar. The powder of SnI_2_ was obtained by grinding the SnI_2_ beads with a pestle and a mortar. SnO_2_ powder was synthesized in-house as reported.^27^ Toluene (anhydrous, 99.8%), dimethylformamide (DMF, anhydrous, 99.8%), dimethyl sulfoxide (DMSO, anhydrous, ≥99.9%), and anisole (anhydrous, 99.7%) were purchased from Merck-Sigma-Aldrich.

### Synthesis

Quartz substrates were cleaned by ultrasonic bath (5 min in acetone + 5 min in isopropanol) and UV-ozone treatment for 10 min. In a glovebox with low levels of O_2_ ≲ 0.5 ppm and H_2_O ≃ 0.8 ppm, two parent solutions (1.55 M) of pure Pb-based and pure Sn-based perovskites (Cs_0.25_FA_0.75_PbI_3_ and Cs_0.25_FA_0.75_SnI_3_) were prepared by stirring overnight the specific perovskite precursors in DMF and DMSO with a volumetric ratio of 4:1. Moreover, a solution of SnF_2_ (0.5 M) was prepared by stirring overnight SnF_2_ powder in DMSO and stirring it again for 15 min at 50 °C the following day. The solution of Cs_0.25_FA_0.75_Sn_0.5_Pb_0.5_I_3_ perovskite was obtained by mixing equal volumes of the two parent solutions and different volumes of SnF_2_ solution. After mixing for 1 h and 30 min, the mixed Sn–Pb perovskite thin films with varying SnF_2_ mol % w.r.t. to SnI_2_ in solution were deposited by antisolvent spin-coating. The perovskite solutions were dripped evenly onto the substrate and spin-coated with an initial rotational acceleration ramp of 500 rpm s^–1^ and a final speed of 3000 rpm for 60 s. After 50 s from the beginning of the rotation, 200 μL of anisole (antisolvent) were poured gently but firmly in ≤1 s from approximately 1–1.5 cm above the surface of the sample. Lastly, annealing at 100 °C for 10 min was performed immediately afterward. The final thickness of the perovskite thin films is ∼250 nm on average, as measured by a profilometer. Reference SnO_2_, SnF_2_, and SnF_4_ thin films were also deposited on quartz substrates for XPS measurements. Each compound powder was individually mixed in DMSO and stirred for 1 h and 30 min. The SnF_2_ and SnF_4_ mixtures were stirred for an additional 15 min at 50 °C to enhance dissolution, resulting in SnF_2_ and SnF_4_ solutions (each 0.5 M). Conversely, the SnO_2_ powder remained dispersed in DMSO. These mixtures were then used to deposit SnO_2_, SnF_2_, and SnF_4_ thin films by spin-coating. Each mixture was dripped evenly onto the substrate and spin-coated with an initial rotational acceleration ramp of 500 rpm s^–1^ and a final speed of 1000 rpm for 40 s. Lastly, annealing at 100 °C for 2 min was performed immediately afterward.

### Steady-State Microwave Conductance (SSMC)

SSMC measurements to study the dark conductivity, i.e., the doping level, of the perovskite thin films were performed in the dark and under N_2_. The microwaves (frequencies between 8.2 and 12.2 GHz) pass through the film located in the microwave cavity cell partially closed with an iris. At the resonant frequency (∼8.5 GHz), a standing wave forms in the cavity, and the maximum of the microwave electric field overlaps with the film. The microwaves are partially absorbed due to the interaction with free, mobile charge carriers and partially reflected. This causes a loss of microwave power (Δ*P*), resulting in a dip at the resonant frequency in the microwave frequency scan.^28,29^ The dip is expressed in *R*_0_ and denotes the fraction of reflected microwave power in comparison to that of a fully reflecting end plate. The normalized microwave power loss signal (Δ*P*/*P*), i.e., the resonant frequency dip, can be simulated to calculate σ_dark_. For more details, see E/M 1. The SSMC measurements are reliable and reproducible because of the fixed sample positioning, microwave cavity dimensions, and iris size, which keep the coupling and quality factor constant. The error estimation is ±∼1% for multiple measurements performed on the same sample and ±∼5% for measurements performed on several samples of the same deposition.

### Time-Resolved Microwave Conductivity (TRMC)

TRMC measurements were performed to study the charge carrier dynamics and transport properties of the perovskite thin films. A pulsed Nd:YAG laser is used to excite charge carriers in the films by pulses of the duration of ∼3.5 ns at a repetition of 10 Hz and a wavelength of λ = 800 nm. The laser intensity is tuned between 10^10^ and 10^13^ photons cm^–2^ by using an array of neutral density filters. During a TRMC measurement, the microwaves pass through the perovskite film mounted in a microwave open cell without the iris (which features an instrumental response time of 2 ns), where they are partially absorbed due to the interaction with free, mobile photogenerated carriers. A circulator separates the incident from the reflected microwaves, and the loss in microwave power between the reflected and the incident microwave is recorded as a function of the time elapsed after the laser pulse (Δ*P*(*t*)). This is related by the sensitivity factor (*K* = 1000 for the microwave open cell) to the time-resolved change in photoconductance between the dark and after illumination (Δ*G*(*t*)), i.e., the transient photoconductance signal. The maximum TRMC signal, normalized by the intensity of the laser, *I*_0_, the absorbed fraction of light at the excitation wavelength, *F_A_*, and a microwave cell form factor, β, can be expressed by the product of the charge carrier yield, φ, and gigahertz-frequency mobilities sum. We assumed φ = 1 for direct bandgap perovskites with a low exciton binding energy at room temperature. It follows that Δ*G*_max_/β*eI*_0_ *F*_*A*_ = Σμ.^28,30^ For more details, see E/M 1. For TRMC, the error estimation is ±∼5% for both multiple measurements performed on the same sample and measurements performed on several samples of the same deposition.

### UV–Vis-NIR Spectroscopy (UV–Vis)

The optical properties (absorption and transmission) of the films were measured with a PerkinElmer LAMBDA 1050+ UV/vis/NIR spectrophotometer with a 150 mm integrating sphere. The absorption (optical density, O.D.) of solutions was measured by a PerkinElmer LAMBDA 365 UV/vis spectrophotometer by using quartz cuvettes with an optical pathway of 0.20 cm.

### Profilometry

The average thickness of the thin films was determined by measurements performed with a Veeco/Bruker Dektak 8 Stylus Profilometer with a stylus tip diameter of 12.5 μm and a force (load) of 5 mg (≃50 μN).

### X-ray Diffraction (XRD)

The XRD analysis of the films was carried out by using a Bruker D8 Advance-ECO X-ray diffractometer equipped with a Cu–K_α_ X-ray source (λ = 1.542) operating at 40 kV and 25 mA and a Lynxeye-XE-T 1D position-sensitive energy-discriminative detector. The measurements were carried out in Bragg–Brentano geometry with a fixed sample illumination of 5.0 mm for a range of angles 2θ = 5°–60°, a step size of 0.01°, and a measuring time of 0.01 s/step.

### X-ray Photoelectron Spectroscopy (XPS)

The elemental composition and chemical state analyses of the films were carried out by using a Thermo Scientific K-Alpha system for XPS, incorporating an X-ray gun based on an Al K_α_ radiation source with an energy of 1486 eV and a spot size kept at the default value of 800 × 400 μm^2^. The samples were transferred into the XPS setup by means of a vacuum transfer module containing the sample stage for XPS measurements, specifically designed for the load lock chamber of the XPS system. The samples were mounted in this transfer module inside the glovebox. Then, the transfer module was accurately sealed and moved to the XPS load lock chamber, used for the automatic transfer of the sample stage in the XPS measurement chamber. All measurements were conducted under high vacuum conditions (*p* < 4 × 10^–7^ mbar). A flood gun operating at 0.15 mA and 1 V was used to replenish the electrons emitted from the sample surface to hinder charging during the measurement. The chemical state analysis of surface XPS scans was performed prior to any etching to avoid damage by the Ar^+^ sputter gun. The XPS peaks were rescaled to the reference peak at *E*_b_ ∼ 284.8 eV in the XPS surface analysis for the C 1s core levels, corresponding to the adventitious C–C chemical state. There were no contributions to the surface XPS scans of the Sn–Pb perovskite films from the underlying quartz substrates, as shown in Figure S16. Depth profiling was conducted by etching the thin film with an argon-based ion beam with an energy of *E* = 1 keV and analyzing its elemental composition after each etching step. While the films suffer from charging during etching, it was still possible to reliably fit the XPS peaks by Advantage software and obtain the compositional depth profiles. We underline that etching limited the detection of organic cations, probably due to preferential sputtering/outgassing of organohalides or low resolution of our measurements. For XPS measurements performed on different samples, the error in the atomic % derived by depth profiling is acceptable for the broad discussion about the elemental variations across the perovskite layers.

### Scanning Electron Microscopy (SEM)

A JEOL JSM-IT700HR field effect scanning electron microscope was used to obtain top-view images of the films and analyze their elemental composition. SEM images were obtained by probing secondary electrons (SE) with an Everhart–Thornley (ET) type SE detector for high-vacuum observation in the chamber, operating the SEM at 3 kV and 30 pA.

In detail, we show in Figure S7 that no degradation of the crystallized perovskite films occurs in the N_2_-filled glovebox on the time scale of days. Nevertheless, the crystallized perovskite films were analyzed as soon as possible after each deposition. All SSMC and TRMC measurements were done by sealing the microwave cells under N_2_ in the glovebox and performed in ∼2 days after each deposition. All XPS measurements were carried out by using a vacuum transfer module specifically designed for the XPS system. For all the measurements not performed under N_2_ or vacuum, to minimize the effect of the exposure to ambient air, the absorption of solutions (placed in cuvettes closed with a cap and sealed with Parafilm) was immediately measured after bringing them out of the glovebox, and the perovskite thin films were transferred to the characterization setups by means of an airtight sample holder and immediately measured after being removed from it.

## Results and Discussion

First, we aim to quantify the extent of tin oxidation in the SnI_2_ precursor used to synthesize mixed Sn–Pb perovskites. SnI_2_ is commercially available in the form of beads that are ground before usage. These SnI_2_ beads are stored in a N_2_-filled glovebox, with parts per million levels of oxygen and moisture. However, the residual oxygen and other chemicals in the glovebox, such as iodine,^31^ could oxidize SnI_2_, especially in powder form. In this experiment, we varied the storage period of the SnI_2_ in the glovebox from less than 2 weeks to more than 2 months. We labeled these differently aged SnI_2_ precursors as new, aged, and strongly aged, respectively. As reported in literature,^31,32^ SnI_4_ readily dissolves in toluene, but SnI_2_ does not. Hence, the differently aged SnI_2_ precursors were mixed in anhydrous toluene to extract any formed SnI_4_. After the mixture was stirred overnight, the undissolved SnI_2_ was filtered, and the yellow-colored toluene solution was measured by UV–vis, as shown in Figure 1. As a reference, we also measured the absorption of SnI_4_ dissolved in toluene, yielding an extinction coefficient of 9545 M^–1^ cm^–1^ (see C 2). From the clear overlap of the spectra with an absorption maximum at λ ∼ 365 nm, we can conclude that indeed, oxidation has occurred. This absorption peak may be due to a ligand-to-metal electronic transition, specifically from HOMO localized on the p-orbitals of iodide atoms to LUMO, which is an Sn–I orbital.^33^ The probable reaction between SnI_2_ and the residual O_2_ is given in eq 2.

2 $$2SnI_{2}+O_{2}\to SnI_{4}+SnO_{2}$$

Furthermore, we noticed that the longer the aging time of the SnI_2_ precursor, the higher the absorption peak, i.e., the fraction of SnI_4_ in the toluene solution and hence in the SnI_2_ precursor. The corresponding fractions, calculated from the optical measurements and provided in Table 1, range from 0.012% to 0.032%.

**Table 1:** Concentration of SnI_4_ in Toluene Solutions, Obtained by Washing in 1.0 mL of Toluene and Filtering of ∼289 mg of Differently Aged SnI_2_ Precursors, and Fraction of Oxidized Sn^4+^

Washed SnI_2_	[SnI_4_] (mM)	Fraction Sn^4+^ to Sn^2+^ (%)
New	0.09	0.012
Aged	0.15	0.020
Strongly aged	0.25	0.032

Interestingly, on adding some smaller lumps of SnF_2_ to the toluene solution, SnI_4_ is reduced back to SnI_2_ leading to a decoloration of the solvent, as shown in Figure 1. This means that SnF_2_ can effectively scavenge oxidized Sn^4+^ (in the form of SnI_4_), as given by eq 1.^23^ In short, we conclude that the susceptibility of SnI_2_ to oxidation is a key factor limiting the quality of the SnI_2_ precursor and, consequently, of the resulting mixed Sn–Pb perovskite thin films.

**Figure 1:**
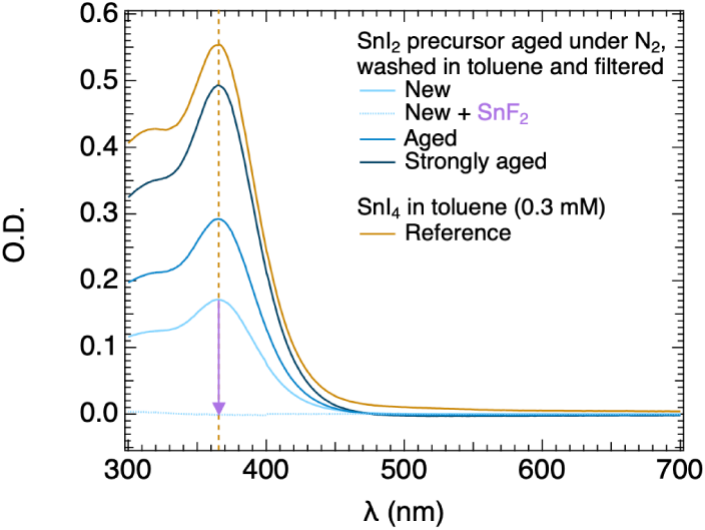
Absorbance spectra of toluene solutions obtained by aging the SnI_2_ precursor in a glovebox for different periods, extraction, and filtering. The spectra, recorded in a 0.20 cm-thick cuvette, are compared to the reference absorption spectrum of SnI_4_ dissolved in toluene (in yellow). In addition, the effect of the SnF_2_ addition (purple arrow) on the optical absorption is also shown (dotted line).

Next, we investigated the effects of tin oxidation and the counteracting effect of SnF_2_ on the final crystallized perovskite layers. In a new experiment, we deposited Cs_0.25_FA_0.75_Sn_0.5_Pb_0.5_I_3_ using SnI_2_ aged for different periods (0, 2, and 20 days). SnI_2_ was dissolved together with the other precursors in a mixture of DMF and DMSO. The SnF_2_ concentration was varied by adding different volumes of a concentrated SnF_2_ stock solution to the perovskite precursor solution to ultimately obtain 0, 1, 2, 5, 10, and 20 mol % SnF_2_ with respect to the ideally present amount of SnI_2_ in such a solution. The final unfiltered solutions were directly used for spin-coating the perovskite thin films.

We first studied the optical, structural, and morphological properties of the perovskite layers with varying concentrations of SnF_2_. The optical absorption spectra measured by UV–vis, shown in Figure S9, show no significant changes with varying SnF_2_ additions. The bandgap energy is *E*_*g*_ = 1.26–1.27 eV, in line with the literature for similar perovskite compositions.^13,34–36^ The XRD patterns, full width at half maximum of the XRD peaks, and crystal lattice parameters given in Figure S10 do not show major differences. Similarly, the morphology of the films also exhibited little difference with varying SnF_2_ additions, as shown in the top-view SEM images in Figure S18.

Then, we studied to what extent Sn^4+^ in the perovskite precursor solution affects the doping level, i.e., the dark conductivity, σ_dark_, of the final crystallized perovskite layers. On top of that, we investigated how the introduction of SnF_2_ in such a solution mitigates the presence of Sn^4+^, again by studying σ_dark_.

To investigate the σ_dark_ of the perovskite thin films, we employed steady-state microwave conductance (SSMC) measurements. This technique allows the determination of σ_dark_ without using electrodes, thanks to the interaction of microwaves and mobile charge carriers. To measure σ_dark_, the film is placed in N_2_ in a microwave cavity cell. By sweeping across the microwave regime, the resonant frequency can be determined, at which a standing wave is formed in the cavity comprising one full oscillation (∼8.5 GHz) and the maximum of the microwave electric field overlaps with the film, as shown in Figure 2a. As a result, a dip in the microwave reflection frequency scan appears. An increase in σ_dark_ leads to an enhancement of the microwave absorption and thus a reduced reflection, resulting in a deepening of the resonance frequency dip. The dip is expressed in *R*_0_ and denotes the fraction of reflected microwave power in comparison to a fully reflecting end plate. In short, the deeper the dip, the higher the σ_dark_. More information about the SSMC technique is in E/M 1.^28,29^

**Figure 2:**
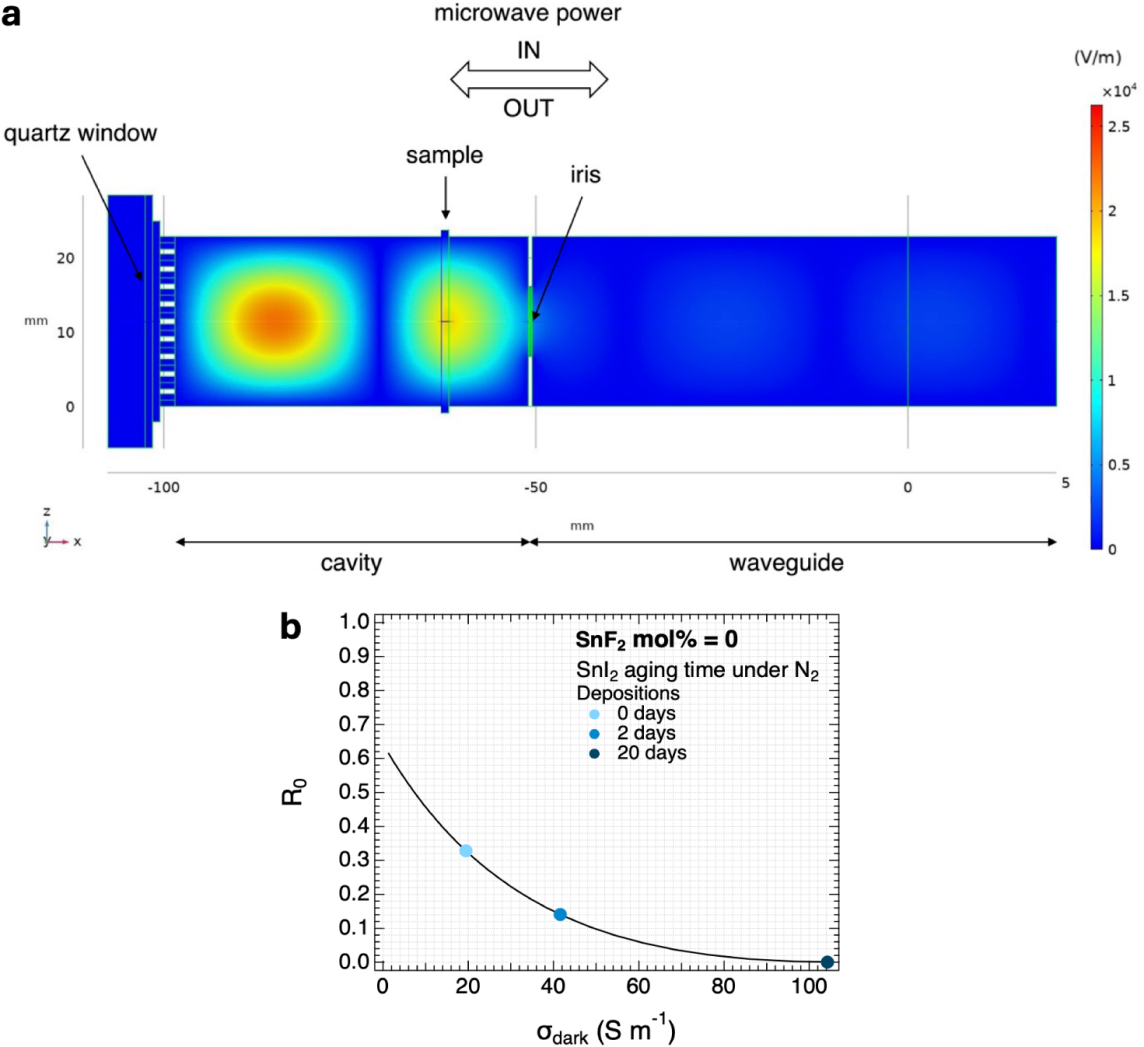
(a) Simulation based on finite element method analysis of the magnitude of the total microwave electric field and its distribution in the cavity cell, longitudinal side-view. (b) Calibration curves relating *R*_0_ of the resonant frequency dips in the SSMC frequency scans to the σ_dark_ of the perovskite thin films. The colored data points correspond to the *R*_0_ and σ_dark_ values for perovskite thin films with 0 mol % SnF_2_ belonging to the depositions shown in Figure 3a–c.

To quantify σ_dark_ from the resonant dip in an SSMC frequency scan, we developed a model by using a computational finite element method (COMSOL Multiphysics).^37^ With this software, our microwave cavity was modeled, as represented in Figure 2a.^37^ The model takes into account the dimensions and relevant dielectric properties of the materials in the cavity, loaded with a sample. By numerically solving the Maxwell equations in each finite element in the cavity, the microwave reflection as a function of frequency and of the σ_dark_ of the sample can be calculated. The simulated resonant characteristics are compared to the experimental results obtained by SSMC measurements to verify the quality of such modeled fits. The magnitude of the microwave electric field and its distribution in the loaded cavity cell are shown in Figure 2a. Finally, a calibration curve relating *R*_0_ and σ_dark_ is derived, allowing us to retrieve σ_dark_ from the dip, as shown in Figure 2b (more details in M 1).^37^

For obtaining σ_dark_ with more precision from Figure 3a, the effect of the specific quartz substrates on which the perovskite film was deposited was taken into account (more details in M 1). For 0-days-aged SnI_2_, we observe only for the sample with 0 mol % SnF_2_ a clear dip deepening. For this series, even an addition of 1 mol % SnF_2_ is sufficient to reduce the dip deepening to a σ_dark_ level close to our detection limit. For the 2-days-aged but definitely also for the 20-days-aged SnI_2_, respectively in Figure 3b,3c, more SnF_2_ is required to reduce the dip deepening. The corresponding σ_dark_ values are extracted using Figure 2b from the dips, and the results are shown in Figure 3d. Clearly, the longest-aged SnI_2_ shows the highest σ_dark_ values in the absence of SnF_2_, reaching values >100 S m^–1^. However, the introduction of 2 mol % SnF_2_ is sufficient to reduce σ_dark_ from ∼104 to ≲2.6 S m^–1^. Despite some fluctuations in the minimum value of σ_dark_, we did not observe any appreciable change in σ_dark_ for higher mol % SnF_2_. Therefore, we conclude that, depending on the initial oxidation of the SnI_2_, an addition of 1 to 2 mol % SnF_2_ is sufficient to suppress doping in mixed Sn–Pb perovskite thin films and that a larger SnF_2_ concentration seems superfluous. Moreover, the minimum SnF_2_ addition required to significantly reduce doping is not absolute, but it is highly dependent on the initial oxidation level of the SnI_2_ precursor.

**Figure 3:**
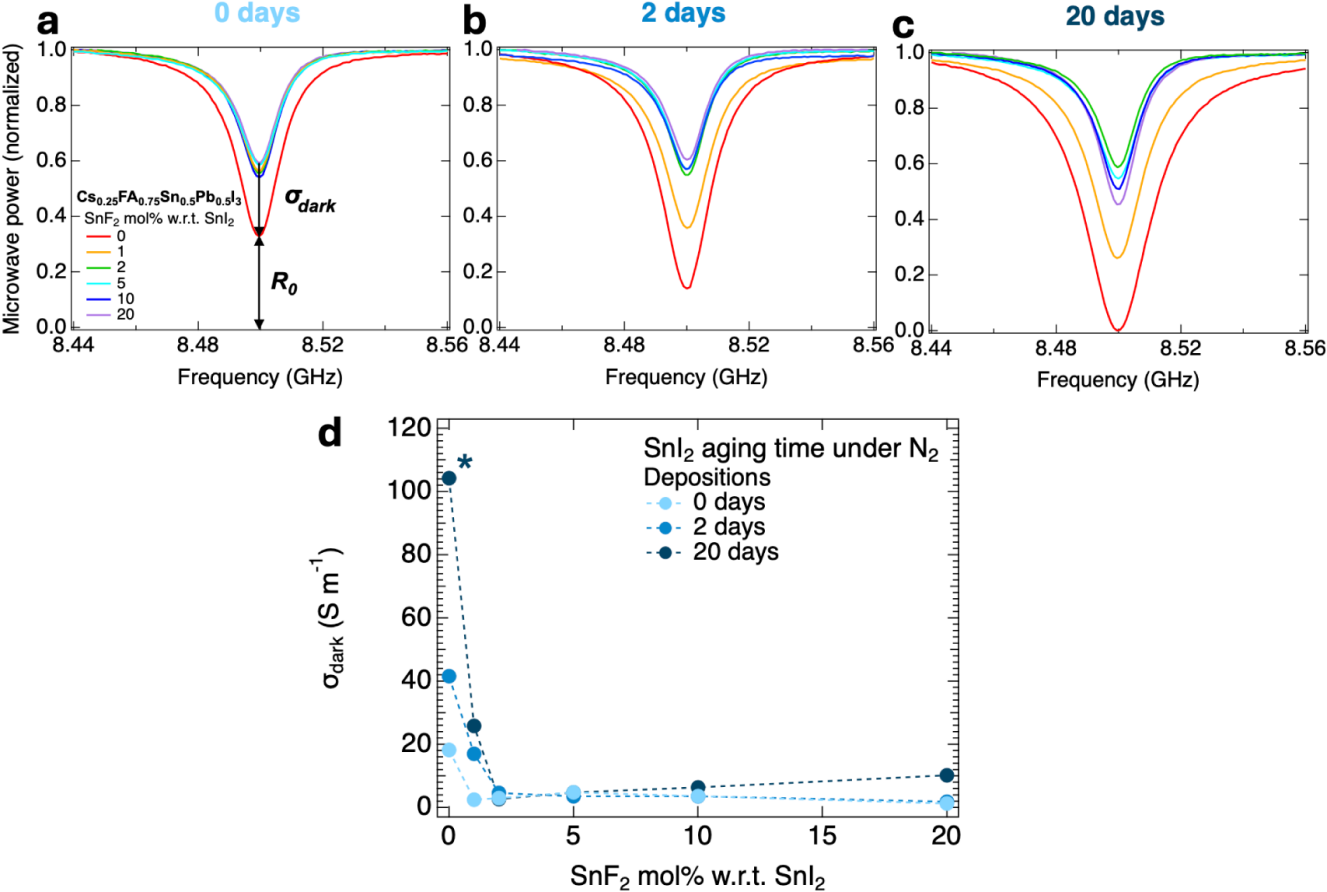
(a–c) SSMC frequency scans of perovskite thin films belonging to different depositions made with SnI_2_ precursor of different purity, i.e., aged for 0 days, 2 days, and 20 days in a glovebox, and varying SnF_2_ additions, showing the change in σ_dark_ and *R*_0_. (d) Effect on σ_dark_ of differently aged SnI_2_ precursor and varying SnF_2_ concentrations. The marker (*) next to a data point indicates lower accuracy in the determination of σ_dark_, as the resonant dip for the corresponding layer is close to the upper detection limit of the SSMC technique.

Next, we studied the charge carrier dynamics in the perovskite thin films prepared with a 0-days-aged SnI_2_ precursor, with varying SnF_2_ concentrations, by time-resolved microwave conductivity (TRMC). The TRMC technique is based on generating excess charge carriers in a perovskite layer loaded in a microwave cell under N_2_ by means of a nanosecond pulsed laser. Note that this method only measures changes in conductivity (AC technique), and the response time of the used microwave open cell amounts to 2 ns. More details about the TRMC technique are in E/M 1.^28,30^ Excitation was carried out at a wavelength of λ = 800 nm, and the laser intensity was varied to induce different photoinduced carrier densities.

A comparison between the TRMC traces for the perovskite thin films with varying SnF_2_ additions is shown in Figure 4a. The TRMC traces show a rapid increase in the photoconductance at the beginning of the photoexcitation, followed by a decay due to simultaneous charge carrier immobilization in traps and recombination via different pathways. The maximum TRMC signal at the lowest intensity is linked to the product of the electron and hole mobility sum, Σμ, and the photoconversion yield, φ.^28,30^ We observed in Figure 4a that the maximum TRMC signal does not change much as a function of the SnF_2_ concentration, except for the lower signal of the perovskite layer with 0 mol % SnF_2_. Considering the higher σ_dark_ of this sample, we ascribed the lower signal to the rapid recombination with dark free holes occurring within the experimental time resolution, which results in an apparent lower signal. Hence, we assumed that all samples present the same mobility sum, Σμ = 32 cm^2^ V^–1^ s^–1^, irrespective of the SnF_2_ concentration, which is in line with the observations from the UV–vis, XRD, and SEM results, and with other reported values.^34,38^

**Figure 4:**
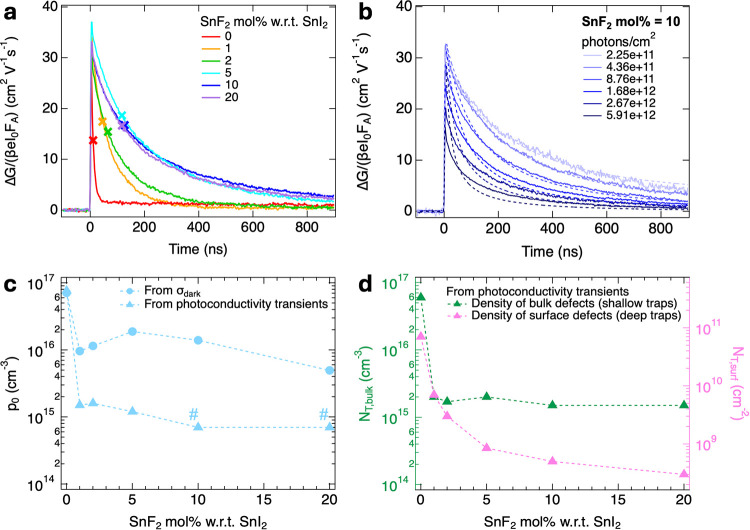
(a) Comparison between TRMC traces of perovskite thin films with varying SnF_2_ additions, belonging to the best-performing deposition in Figure 3a. The traces were measured at the same intensity of ∼6–7 × 10^11^ photons cm^–2^. The colored cross markers indicate the time to reach half of the initial maximum photoconductivity signal, used as a metric of the carrier lifetimes. (b) Intensity- and time-dependent TRMC traces for a perovskite film with 10 mol% SnF_2_. The solid lines represent the experimental traces obtained by using a microwave OC, while the dashed lines correspond to the modelled traces resulting from the 1D drift-diffusion simulator. Values of (c) *p*_0_ (in light blue) and (d) *N*_T_, distinguishing *N*_T,bulk_ (in dark green) and *N*_T,surf_ (in pink), as a function of the SnF_2_ concentration. The data points for *p*_0_ indicated by dot markers are calculated from the corresponding σ_dark_ values obtained by fitting the SSMC frequency dips in Figure 3a. The data points for *p*_0_ and *N*_T_ indicated by square markers are obtained by the fitting of the time- and laser-dependent TRMC traces in Figure S8. The marker (#) next to a data point indicates that the shown value of *p*_0_ is an upper limit derived from the drift-diffusion simulations of the TRMC traces.

According to the literature, the effective masses of electrons and holes are similar for mixed Sn–Pb perovskites.^20^ For this reason, we assume that μ*_h_* ≈ ∑μ/2. Knowing μ*_h_* allows us to calculate the concentration of dark free holes, *p*_0_, from σ_dark_ by using σ_dark_ = *e*μ*_h_p*_0_, where *e* is the elementary charge. The *p*_0_ values as a function of the SnF_2_ concentration for the deposition prepared with a 0-days-aged SnI_2_ precursor are shown in Figure 4c and will be discussed later on. The *p*_0_ values for all depositions made by using a differently aged SnI_2_ precursor for varying SnF_2_ concentrations are provided in Figure S6.

To obtain a better understanding of the underlying processes governing the charge carrier dynamics, the time- and laser light-dependent TRMC traces were fitted with SIMsalabim. This is a 1D drift-diffusion simulator for semiconductor materials, where the coupled set of continuity equations with the Poisson equations is numerically solved. It includes the photogeneration of both electrons and holes, their recombination and trapping, and the effect of localized ions and dopants. Moreover, the simulator allows one to include surface and bulk defect states and set their position within the bandgap, allowing for the distinction between shallow and deep traps. More information about SIMsalabim is provided in M 2.^39^ Using SIMsalabim, the time-dependent TRMC traces were simulated by performing a global fit for all light intensities simultaneously. The modeled TRMC traces resulting from the simulations are shown in Figure 4b for a perovskite film with 10 mol % SnF_2_ (see Figure S8 for the other SnF_2_ additions). The resulting kinetic parameters associated with the best-fit simulated TRMC traces are collected in Table 2. In addition, the found *p*_0_ values are added to Figure 4c, while trap densities are plotted in Figure 4d, both as a function of added SnF_2_.

**Table 2:** Fitted Parameters of the 1D Drift Diffusion Modelling of the TRMC Traces of Perovskite Thin Films with Varying SnF_2_ Concentrations

mol % SnF_2_	0	1	2	5	10	20
*k*_2_ (cm^3^ s^–1^)	9.0 × 10^–10^	6.2 × 10^–10^	6.0 × 10^–10^	6.0 × 10^–10^	5.0 × 10^–10^	4.0 × 10^–10^
*N*_T,bulk_ (cm^–3^)	6.0 × 10^16^	2.0 × 10^15^	1.7 × 10^15^	2.0 × 10^15^	1.5 × 10^15^	1.5 × 10^15^
*N*_T,surf_ (cm^–2^)	7.0 × 10^10^	7.0 × 10^9^	3.0 × 10^9^	8.5 × 10^8^	5.0 × 10^8^	3.0 × 10^8^
*p*_0_ (cm^–3^)	7.6 × 10^16^	1.5 × 10^15^	1.6 × 10^15^	1.2 × 10^15^	<7.0 × 10^14^	<7.0 × 10^14^

Figure 4c collects the *p*_0_ values obtained by fitting the TRMC traces and the *p*_0_ values obtained by the SSMC measurements. For no added SnF_2_, the highest *p*_0_ values are found, while on adding SnF_2_, the *p*_0_ values decrease substantially. The discrepancies between the *p*_0_ values for >1 mol % SnF_2_ obtained by both methods originate from the assumption that the dip deepening of the perovskite layers in the SSMC measurements are exclusively from free, mobile carrier absorption (p-doping). This leads typically to an overestimation of σ_dark_ when close to the SSMC detection limit (more details in M 1). The *p*_0_ values for the 10 and 20 mol % SnF_2_ obtained from fitting of the TRMC traces are upper limits as well. Actually, any value taken below this threshold results in identical modeled TRMC traces. This means that *p*_0_ has reached a sufficiently small value to no longer influence the carrier dynamics of the perovskite film. Hence, only an upper limit can be given in this case. Nevertheless, we found that both methods show the same trend for *p*_0_, where 1 mol % SnF_2_ yields the strongest reduction in doping with no further decrease for higher SnF_2_ concentrations.

Figure 4d shows the density of trap states, *N*_T_, obtained by fitting the TRMC traces as a function of the SnF_2_ concentration. More specifically, the TRMC simulations allowed us to obtain the trap state density in the bulk, *N*_T,bulk_, and at the surface, *N*_T,surf_, of the perovskite thin films.

*N*_T,bulk_ are shallow trap states, while *N*_T,surf_ are deep states. The positions of both types of trap states in the bandgap slightly change with higher SnF_2_ concentrations (see Table S1). Various combinations of deep and shallow trap states for bulk and surface defects were tested, but these did not yield accurate results, further validating the obtained simulations.

The value of *N*_T,bulk_ decreases by more than an order of magnitude to ∼2 × 10^15^ cm^–3^ on introducing 1 mol % SnF_2_ but does not reduce further for higher SnF_2_ concentrations up to 10 mol %, very similar to *p*_0_. Thus, on the introduction of 1 mol % SnF_2_ specifically, the bulk perovskite lattice improves, yielding longer charge carrier lifetimes. This can be explained by the reduction of the pseudomonomolecular recombination of excited electrons with the dark free holes, in line with other reports.^12,13,34^ On the other hand, *N*_T,surf_ keeps decreasing with higher SnF_2_ addition, showing a reduction of over 2 orders of magnitude to 3 × 10^8^ cm^–2^ for 10 mol % SnF_2_. Moreover, the lifetimes increase by more than 1 order of magnitude from ∼10 to ∼130 ns when going from 0 to 10 mol % SnF_2_, as seen in Figure 4a. Hence, we believe that the reduction of *N*_T,surf_ (surface defects, deep traps) and associated trap-assisted recombination by SnF_2_ is linked to the doubling of the carrier lifetimes. We think that *N*_T,surf_ is originated by surface-stable Sn^4+^ defects, as reported in the literature,^10^ and that the removal of Sn^4+^ by SnF_2_ suppresses these defects and leads to the observed increase in carrier lifetimes.

At this point, we want to link the Sn^4+^ concentration in solution to the dark free hole concentration, *p*_0_ in the perovskite film (i.e., to the Sn^4+^ concentration in the crystal) without added SnF_2_. For this, we first analyzed a slightly aged SnI_2_ precursor by absorption spectroscopy as shown in Figure S5a, yielding a fraction of Sn^4+^ to Sn^2+^ in solution of 0.013% as given in Table S2. Then, we used an identical SnI_2_ precursor to prepare a spin-coating solution and deposit a perovskite film without added SnF_2_. From the measured σ_dark_, we calculated *p*_0_ amounting to 6.6 × 10^16^ cm^–3^ (see Figure S5b). Considering that *p*_0_ corresponds to half the concentration of Sn^4+^ in the perovskite film and that the density of tin atoms in the perovskite crystal is ∼2 × 10^21^ cm^–3^, this means that ∼0.0016% of the tin atoms are involved in doping. From this ratio, it is inferred that ∼12% of the Sn^4+^ in the perovskite solution leads to doping in the perovskite layer (see C 3). We believe that this number is due to the limited intake of SnI_4_ into the perovskite structure during the crystallization process, while the major part is removed with the excess solution lost during spin-coating.

From the above, for the depositions made with differently aged SnI_2_ precursors in Figure 3 we can couple the initial Sn^4+^ concentration in solution to *p*_0_ of the perovskite films without added SnF_2_ (for details about *p*_0_ and the Sn^4+^ concentration in these crystallized films, see Table S3 and C 4). In view of the fact that only ∼12% of the Sn^4+^ in the perovskite solution leads to doping, we calculated the initial concentration of Sn^4+^ (in the form of SnI_4_) in solution, as well as the corresponding fractions with respect to the SnI_2_ precursor. The results are given in Table 3.

**Table 3:** Initial Concentration of Sn^4+^ in Solution for the Depositions Made with Differently Aged SnI_2_ Precursors and Corresponding Fractions of Sn^4+^ to Sn^2+^

Aged SnI_2_ precursor	[SnI_4_] (mM)	Fraction Sn^4+^ to Sn^2+^ (%)
0 days	0.06	0.015
2 days	0.13	0.033
20 days	0.32	0.083

Now we can calculate the excess concentration of SnF_2_ required to suppress doping in the perovskite films. We deduced that 1 mol % SnF_2_ is sufficient to reduce the σ_dark_ for the 0-days-aged SnI_2_. This means that ∼70 times excess of SnF_2_ over the initial concentration of Sn^4+^ in the spin-coating solution is needed to push Reaction (1) in eq 1 to the right (see C 5 and Table S4). For the more oxidized 20-days-aged SnI_2_ precursor, 2 mol % SnF_2_ is needed, corresponding to a similar excess of SnF_2_. With higher SnF_2_ concentrations, Reaction (1) in eq 1 is more complete, leading to the removal of the final traces of Sn^4+^, reducing *N*_T,surf_ and more than doubling the charge carrier lifetimes of the perovskite layers. Furthermore, when comparing the decrease in *p*_0_ of just an order of magnitude, from 7.1 × 10^16^ cm^–3^ to 5.0 × 10^15^ cm^–3^ going from 0 to 20 mol % SnF_2_, to the density of tin atoms in the perovskite crystal of ∼2 × 10^21^ cm^–3^, we conclude that only a minuscule fraction (∼1 × 10^–4^) of the perovskite structure is modified by SnF_2_, contrarily to other works.^24–26^ Hence, studying tin oxidation, doping, and the effect of SnF_2_ on the crystallized perovskite films by analyzing variations in optical bandgap, crystallinity, and morphology is extremely difficult, if not meaningless, since these minuscule changes are below the detection limit of XRD, UV–vis, and cannot be assessed by SEM. This is in line with our previously shown UV–vis, XRD, and SEM results in Figures S9, S10, and S18 and with our previous research about the effect of short- and long-term exposure to oxygen on mixed Sn–Pb perovskite films.^40^ Conversely, significant changes are visible in the electronic properties of the perovskite layers, which are the most sensitive to tin oxidation and doping. Hence, very sensitive optoelectronic and spectroscopic techniques are required to investigate these effects, e.g., the microwave-based techniques such as SSMC and TRMC that we used in this work.

To observe the tin oxidation products and the impact of SnF_2_, we also studied the elemental composition of the films by X-ray photoelectron spectroscopy (XPS) of the perovskite layers prepared from 0-days-aged SnI_2_ precursor and varying concentrations of SnF_2_. We verified the presence of SnO_*x*_ even in the 0-days-aged SnI_2_ by the XPS analysis of the O 1s core levels in Figure S11. We also analyzed perovskite thin films with 0, 2, and 10 mol % SnF_2_ additions. We present in Figure S12 the surface XPS for Cs, Sn, Pb, I, O, and F as a function of the binding energy, *E*_b_, measured for layers with varying SnF_2_ concentrations. Figure 5a shows the surface XPS analysis of the O 1s core levels, revealing that SnO_*x*_ species are formed at the surface upon SnF_2_ addition. Interestingly, from Figure 5c it seems that these SnO_*x*_ species increasingly accumulate on the surface of the layers for higher SnF_2_ concentrations. This is also in line with the XPS surface analysis of the Sn 3d core levels for the same samples presented in Figure S15. From the Wagner plots in Figure S13 derived from the surface XPS measurements, including those for the SnO_2_, SnF_2_, and SnF_4_ reference layers in Figure S14 and Table S5, and constructed following the method reported in the literature,^41^ we attributed the main fitted XPS peak at *E*_b_ ∼ 486.4 eV for the perovskite film with 0 mol % SnF_2_ to Sn^2+^ in the perovskite crystal structure, while for the film with 2 mol % SnF_2_ the main fitted peak at *E*_b_ ∼ 486.5 eV is attributed to Sn^2+^ in the form of SnO, and the other fitted peak at the highest *E*_b_ ∼ 487.4 eV appearing upon SnF_2_ addition is attributed to SnO_2_. This seems also the case for the film with 10 mol % SnF_2_, presenting two fitted peaks at *E*_b_ ∼ 486.6 eV and *E*_b_ ∼ 487.5 eV, respectively. We suppose that SnF_2_ affects the interaction of SnO_*x*_ in solution, causing its deposition on the surface, as also reported in the literature.^25^ Furthermore, the XPS depth analysis for the Sn 3d and I 3d core levels, respectively, shown in Figure 5b,5d (see Figure S17 for other elements), revealed that the highest SnF_2_ addition leads to the strongest Sn-rich/I-poor conditions at the surface. In fact, the Sn:Pb ratio goes from 0.3:0.7 to 0.7:0.3, while the (Sn+Pb):I ratio goes from 1:3.2 to 1:1.7 on going from 0% to 10% SnF_2_ addition, as mentioned in Table S6. This is not only an indication of the accumulation of SnO_*x*_, but it also reveals compositional heterogeneity at the film surface for high SnF_2_ additions.

**Figure 5:**
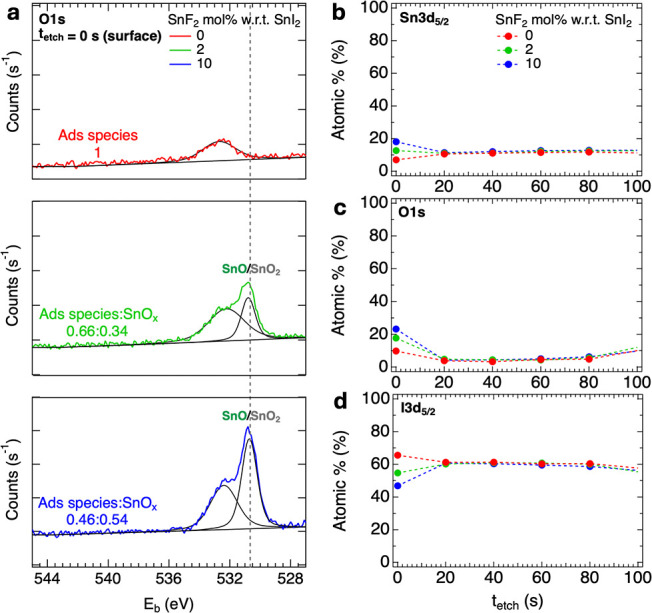
(a) XPS surface analysis and peak fitting showing the O 1s core level and (b–d) XPS depth profiling focusing on the Sn 3d, O 1s, and I 3d core levels of perovskite thin films with 0 (in red), 2 (in green), and 10 (in blue) mol % SnF_2_ additions. In (a), the intensity of the surface XPS signal for the different electron transitions and element orbitals is shown as a function of the electron binding energy, *E*_b_. The chemical state analysis of this surface XPS scan is performed prior to any etching to avoid damage by the Ar^+^ sputter gun. The results from peak fitting are shown (solid lines in black). These were attributed to different oxidation species, whose ratio for each film is indicated as Ads species:SnO_*x*_. The fitted XPS peak located at *E*_b_ = 532.5 ± 0.31 eV and defined as Ads species is likely a collection of narrower XPS peaks corresponding to O-containing adsorbed species, i.e., O–H, O=C, and O–C species (going from low to high *E*_b_) as reported.^25^ In (b–d), the atomic % in the XPS depth profiling is shown as a function of the time of etching through the film, *t*_etch_, and it is shown up to *t*_etch_ = 100 s, which corresponds to tens of nm from the top surface of the film. The depth profiles are represented with markers to highlight the atomic % after each etching step and dashed lines as a guide to the eye.

Scheme 1 summarizes how tin oxidation in the SnI_2_ precursor plays a dominant role in the electronic properties of mixed Sn–Pb perovskites and the impact of SnF_2_. We showed that even in 0-days-aged SnI_2_ a small fraction oxidizes to SnI_4_ and SnO_*x*_. It is anticipated that SnI_4_ dissolves in the perovskite precursor solution, presumably by forming SnI_4_·(DMSO)_2_ complexes,^42^ while solid SnO_*x*_ is dispersed in the solution. Without SnF_2_ addition, a fraction of ∼12% of the SnI_4_ is incorporated in the perovskite thin film during crystallization, while the rest of the SnI_4_ and SnO_*x*_ is most likely lost with the excess spin-coating solution. In the perovskite film, the incorporated Sn^4+^ is displaced toward the surface, leaving $V_{\mathrm{Sn}}^{\prime \prime }$ in the bulk, which is responsible for the formation of dark free holes (p-type doping). Both Sn^4+^ at the surface and $V_{\mathrm{Sn}}^{\prime \prime }$ increase the crystal defect density, leading to deep and shallow traps, respectively. As described in the literature and shown in eq 1,^23^ a ligand exchange reaction occurs in solution that removes the SnI_4_. From our quantitative analysis, a ∼70 times excess of SnF_2_ is required to scavenge most of the Sn^4+^ and to prevent its incorporation in the perovskite film. Besides, SnF_2_ affects the interaction of SnO_*x*_ in the spin-coating solution, causing its deposition on the perovskite surface, contrarily to the deposition without SnF_2_. We think that most of the SnF_4_ is likely excluded from the perovskite crystal lattice and washed away with the excess solution during spin-coating. However, a small part of F^–^ may remain on the surface of the films, as indicated by the surface F 1s orbitals visible for films with ≥10 mol % SnF_2_, as shown in Figure S12f.

**Scheme 1:**
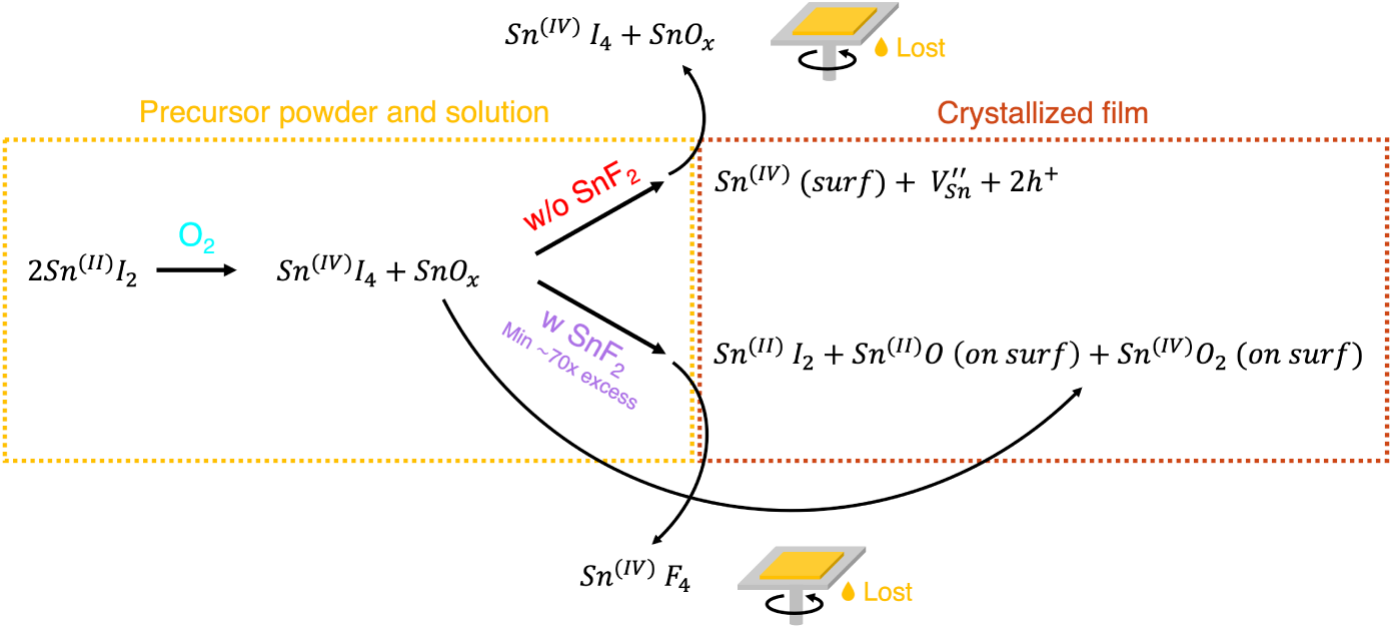
Overview of Reactions Involved with SnI_2_ Precursor Oxidation to SnI_4_ and the Impact of SnF_2_ on the Perovskite Thin Films

These findings emphasize that while SnF_2_ can enhance the electronic properties, such as the charge carrier transport, by reducing *p*_0_ and *N*_T_, it is likely not the definitive solution for improving mixed Sn–Pb perovskite solar cells. The addition of SnF_2_ leads to compositional heterogeneity and the accumulation of SnO_*x*_ at the film surface. Hence, an overly high SnF_2_ addition can make the surface of the perovskite layer more sensitive to post-synthesis oxidation, potentially compromising its stability over time. Additionally, this may lead to band misalignment and/or defects at the interface with the transport layer, hindering carrier transport. Except for a few reports,^32,43,44^ we suggest that future research should focus on methods to improve the purity and storage conditions of the SnI_2_ precursor. Furthermore, combining SnF_2_ with other additives that improve the compositional homogeneity and microstructure could also be a promising strategy to tackle the challenges of mixed Sn–Pb perovskites from multiple angles to ultimately boost the efficiency of the corresponding solar cells.

## Conclusions

We obtained SnI_2_ precursor of different purity by aging in a N_2_-filled glovebox. We noticed that residual oxygen produces SnI_4_ as an oxidation product, which we quantified by means of optical absorption spectroscopy. To study the effects of tin oxidation and the counteracting impact of SnF_2_, we deposited by spin-coating mixed Sn–Pb perovskite thin films with the composition Cs_0.25_FA_0.75_Sn_0.5_Pb_0.5_I_3_. We varied the SnI_2_ precursor purity and the SnF_2_ mol % w.r.t. the SnI_2_ precursor in solution, ranging from 0 to 20 mol %. By applying SSMC, we observed a decrease in dark conductivity from ∼100 to <∼1 S m^–1^ by changing the SnF_2_ concentration from 0 to 1–2 mol %. By fitting the intensity-dependent photoconductivity signals measured by TRMC, we found that both doping and defect density concomitantly decrease with increasing SnF_2_ concentration. This more than doubles the photoinduced carrier lifetimes from ∼10 to ∼130 ns for SnF_2_ concentrations up to 10 mol %, although only a minuscule fraction (∼1 × 10^–6^) of the perovskite film is modified by SnF_2_. Without adding SnF_2_, we inferred that Sn^4+^ is displaced at the film surface, leaving tin vacancies in the bulk that are charge-compensated by dark free holes. Moreover, we found that ∼12% of the Sn^4+^ (SnI_4_) in the perovskite solution leads to doping in the perovskite layer. To scavenge most of Sn^4+^, a minimum of ∼70 times excess of SnF_2_ over the initial concentration of Sn^4+^ in the spin-coating solution is necessary. Hence, the minimum SnF_2_ addition required to reduce doping and crystal defects is not absolute but depends on the initial oxidation of the SnI_2_ precursor. For higher SnF_2_ concentrations, the final traces of Sn^4+^ can be removed, which results in the decrease of surface defects, reduced carrier recombination, and more than doubled lifetimes. The reduced doping of these perovskite films with SnF_2_ addition, in combination with the reduced defect density, makes these perovskite layers ideal candidates for efficient solar cells. However, SnF_2_ also induces compositional heterogeneity and the accumulation of SnO_*x*_ at the film surface, which could potentially have a negative effect on the efficiency of mixed Sn–Pb perovskite solar cells.

## Supplementary Material



## Notes

Experimental section/methods describing SSMC and TRMC techniques (E/M 1); modeling details about SSMC (M 1) and TRMC (M 2) simulations; calculations details about lattice parameter (C 1); molar extinction coefficient (C 2); connection between the fraction of oxidized tin in solution and in the perovskite thin films (doping) (C 3 and C 4); required excess of SnF_2_ additive (C 5); SSMC and TRMC; UV–vis-NIR spectroscopy; XRD, XPS, and SEM results and analysis; Figures S1–S18; Tables S1–S6 (PDF)
