# Supplementary material for: Quantitative Analysis of the Doping and Defect Density in Mixed Sn–Pb Perovskites Mediated by SnF2
Source: Chem Mater. 2025 Oct 2;37(19):7611–21. doi: 10.1021/acs.chemmater.5c00816 (PMC12529774; doi:10.1021/acs.chemmater.5c00816)
Supplement: Supplementary file 1 [file cm5c00816_si_001.pdf]

Quantitative Analysis of the Doping and Defect Density in Mixed Sn-Pb Perovskites Mediated by SnF_2

Authors

Jasmeen Nespoli[†], Maartje J. van der Meer[†], Sander Heester[‡], Jim S. Koning[†], Bart Boshuizen[†], L. Jan Anton Koster[‡], Tom J. Savenije[*,†]

Affiliations

[†]*Department of Chemical Engineering, Faculty of Applied Sciences, Delft University of Technology, 2629 HZ Delft, The Netherlands*

[‡]*Zernike Institute for Advanced Materials, University of Groningen, Nijenborgh 3, 9747AG Groningen, The Netherlands*

Corresponding Author

Tom J. Savenije - E-mail: T.J.Savenije@tudelft.nl

Supporting information (SI)

Experimental section/Methods (E/M)

E/M 1) Steady-state microwave conductance and time-resolved microwave conductivity

The charge carrier properties in the perovskite thin films with varying SnF_2 mol% w.r.t. to SnI_2 precursor in solution was investigated by microwave-based characterization techniques.

The steady-state microwave conductance (SSMC) technique was used to inspect the background conductivity in the dark, σ_{dark}, of perovskite thin films, i.e. the doping level. Each sample under investigation was mounted in a specific microwave cell sealed inside a N_2-filled glovebox. All SSMC measurements were performed in the dark. The microwaves source (frequencies between 8.2-12.2 GHz) is a voltage-controlled oscillator.[2] The microwaves pass through the perovskite thin film located in a microwave cell and they are partially absorbed due to the interaction with free, mobile charge carriers in the dark, and partially reflected. This results in a loss of microwave power (ΔP).[2,3]

The type of microwave cells used for the SSMC measurements is a cavity cell, defined hereafter as CC, partially closed with an iris. During a SSMC measurement, ΔP is probed by a microwave detector and a signal processing system while sweeping across the microwaves frequency range. At the resonant microwave frequency, a standing wave forms inside the cavity, as schematically illustrated in **Figure S1a**. In this condition, the maximum of the microwave standing wave overlaps the sample at approximately ¾ of the cell length, resulting in a highly sensitive measurement of the microwave power loss. This emerges as a dominant dip at the resonant frequency in the microwave frequency scan.[2,3] Moreover, the SSMC frequency scan a fully reflective endplate is also measured. All the SSMC frequency scans of the perovskite layers are compared (normalized) to it. The dip is expressed in R_0 and denotes the fraction of reflected microwave power in comparison to the fully reflecting end plate. The normalized microwave power loss

signal ($\Delta P/P$), *i.e.* the resonant frequency dip, can be related to the total variation in the conductance (ΔG) of the perovskite thin film and simulated to calculate σ_{dark}.

For instance, the SSMC frequency scans of a quartz substrate and a perovskite thin film deposited on the same quartz substrate, normalized by the endplate, can be observed in **Figure S1b**.

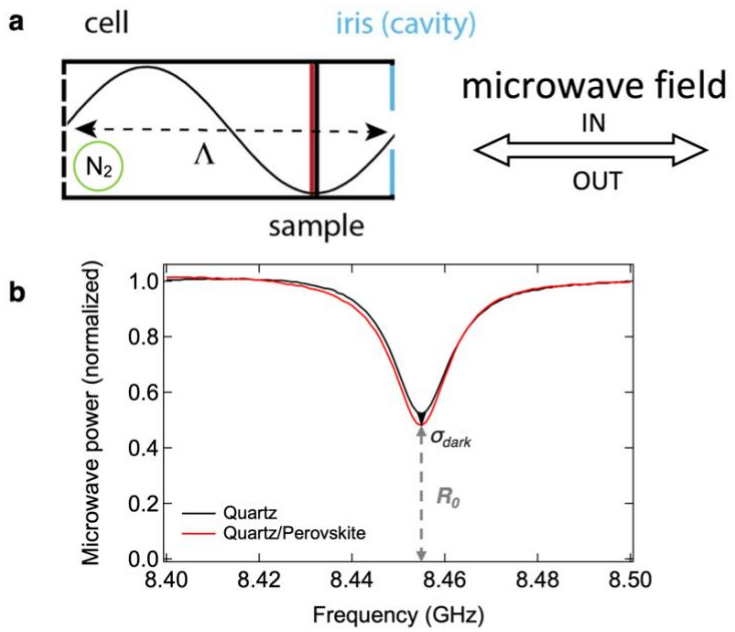

Figure S1: SSMC set-up and measurement. (a) Illustration representing the interaction between the microwaves and sample under investigation in the fully reflective CC of length Λ. The microwave field takes the form of a stationary wave of wavelength Λ at a specific resonant frequency.[2] (b) Frequency scans measured by SSMC and normalized by a fully reflective endplate, showing the results for a quartz substrate and a perovskite thin film deposited on it. The frequency scan of quartz is a reference, since its electrical insulator characteristics define the lower limit of detection in measuring σ_{dark}. By comparing the dips of the quartz substrate and of the perovskite thin film deposited on it, it is possible to attribute the dip deepening of the conductive perovskite layer to σ_{dark} (black arrow). The resonant dip, related to the microwave power reflection coefficient and thus indicated as R_0, is also shown (in grey, dashed double arrow).

The change in conductivity $\Delta\sigma$ measured by SSMC technique derives from the change in charge carrier concentration, Δn, in equilibrium, in the dark at room temperature. As mentioned before, the normalized microwave power loss signal obtained by SSMC can be simulated to estimate σ_{dark} of the sample and then calculate the dark carrier density, n_{dark}, by using **Equation (S1)**.[2]

$$\sigma_{dark} = e\sum \mu n_{dark} = e(\mu_e n_{e,dark} + \mu_h n_{h,dark}) \qquad (1)$$

Where e is the elementary charge and $\mu_{e/h}$ *and* $n_{e/h}$ are, respectively, the mobilities and concentrations of the dark carriers.[2]

In case of perovskite p-type doping, the following **Equation (S2)** is applied.

(2)

$$\sigma_{dark} = e\mu_h p_0$$

In this case, σ_{dark} mainly results from the contribution of only one type of charge carrier and only its mobility needs to be taken into account. The background hole concentration in the dark, p_0, can be calculated using **Equation (S3)**.

$$p_0 = \frac{\sigma_{dark}}{e\mu_h} \quad (3)$$

Where only the mobility of holes, i.e. the majority carrier, is taken into account. When the effective masses of electrons and holes are similar, it is possible to assume that both present the same mobility and thus $\mu_{e/h} = \mu/2$.[2]

Besides, the time-resolved microwave conductivity (TRMC) technique was used to study the charge carrier dynamics and transport properties in the perovskite thin films.

The same working principle and set up described above for the SSMC technique applies to the TRMC technique. The main difference is that the TRMC set-up, schematically illustrated in **Figure S2**,[2] presents an pulsed Nd:YAG laser which is used to generate pulses of the duration of ~ 3.5 ns at a repetition of 10 Hz. The laser pulses excites electrons to the conduction band, leaving holes in the valence band. In TRMC measurements, the light intensity of the excitation laser pulse is tuned between 10^{10} and 10^{13} photons cm^{-2} by using an array of neutral density filters. During a TRMC measurement, the microwaves pass through the perovskite thin film mounted in the microwave cell, where they are partially absorbed due to the interaction with free, mobile photogenerated charge carriers. A circulator separates the incident from the reflected microwaves, which are recorded by a microwave detector and a signal processing system in the form of a reduction in the microwave power (ΔP) between the reflected and the incident microwave, *i.e.* the result of the microwaves absorption by photogenerated charge carriers. This reduction of microwave power is recorded as a function of the time elapsed after the laser pulse ($\Delta P(t)$). The normalized reduction in microwave power is related to the time by **Equation (S4)**.[2,4]

$$\frac{\Delta P(t)}{P} = \frac{P'(t) - P}{P} = -K\Delta G(t) \quad (4)$$

The TRMC measurements were performed by using a microwave open cell, defined hereafter as OC, without an iris. In this case, the microwave passes only one time throughout the sample. As a result, the instrumental response time is reduced to 2 ns compared to 18 ns for the CC, but this is at the expense of a loss of sensitivity, expressed by the K factor, with respect to the CC. The K factor for the OC, K = 1000, was used to correct all TRMC traces.[2]

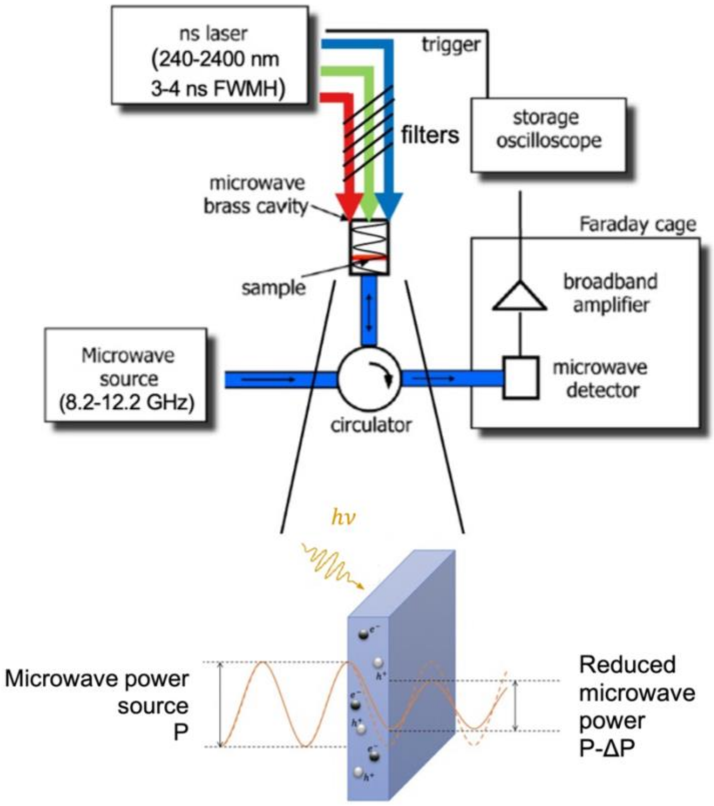

Figure S2: Schematic illustration of the TRMC set-up, showing the interaction between the oscillating microwave electric field and the sample under investigation.[2]

By knowing the *K* factor and the measured microwave power loss as a function of time, the time-resolved change in photoconductance between dark and after illumination can be quantitatively calculated ($\Delta G(t)$). Similarly to **Equation (S1)**, the time-dependent variation in photoconductance is related to the time-dependent variation in electrical conductivity ($\Delta\sigma(t)$), which scales with the time-dependent concentration ($n_e(t)$ and $n_h(t)$, namely the lifetime relative to the recombination rate), and mobilities sum ($\mu = \mu_e + \mu_h$) of free electrons and holes, as shown in **Equation (S5)**.[2,4]

$$\sigma(t) = e \sum \mu n(t) = e(\mu_e n_e(t) + \mu_h n_h(t)) \quad (5)$$

In order to directly compare different samples, the maximum TRMC signal can be expressed by the product of charge carrier yield and gigahertz-frequency mobilities sum. If every absorbed photon generates a single electron-hole pair, which commonly occurs in direct bandgap perovskites with low exciton binding energy at room temperature, the yield of free charge carrier generation (φ) is equal to 1. This is expressed by **Equation (S6)**.[2,4]

$$\phi = \frac{Ln}{F_A I_0} \quad (6)$$

Where I_0 is the intensity of the laser (photons per laser pulse per unit area) and F_A is the absorbed fraction of light at the excitation wavelength. By combining **Equation (S1)** and **Equation (S6)**, the equation relating ΔG_{max}, the charge carrier yield and mobilities sum can be obtained, as shown in **Equation (S7)**.[2]

$$\phi(\mu_e + \mu_h) = \frac{Ln}{F_A I_0}\frac{\Delta\sigma}{en} = \frac{L}{F_A I_0}\frac{\Delta G_{max}}{e\beta L} = \frac{\Delta G_{max}}{F_A I_0 \beta e} \tag{7}$$

The relationship between these important properties can be seen in the transient photoconductance signals, referred to as TRMC traces, in **Figure S3**.

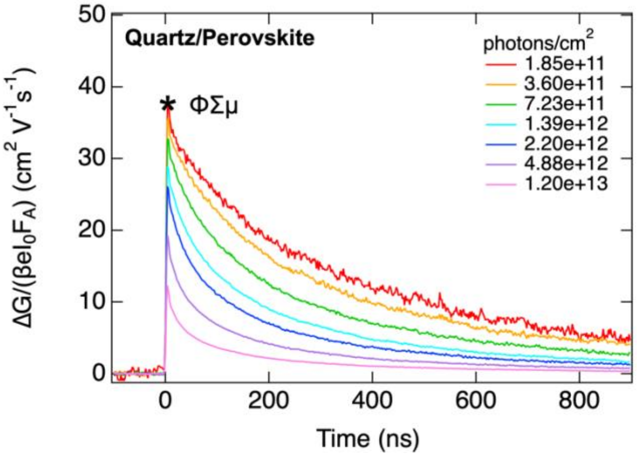

Figure S3: Visualization of the relationship between the maximum variation of photoconductance signal ($\Delta G_{max}/(\beta e I_0 F_A)$) and the product between the free charge carrier generation yield and the mobilities sum ($\varphi\Sigma\mu$) in a typical transient photoconductance signal obtained by TRMC.

Modelling (M)

M 1) Determination of the calibration curves relating R_0, σ_{dark} and the K factor

The calibration curves relating R_0 and σ_{dark} were calculated as follows. The connection between the change in microwave reflected power in the CC, the change in dip deepening (related to R_0) and the change in conductivity is shown in **Equation (S8)**.[5]

$$\frac{\Delta P}{P} = \frac{\Delta R_0}{R_0} = -K\Delta G = -K\Delta\sigma\beta L \tag{8}$$

Where ΔR_0 is the change in dip deepening due to an increase in σ_{dark}, caused by, for instance, doping.

The computational finite element method applied to the CC made possible to study the exact effect of σ_{dark} on R_0 in a (seemingly) continuous manner. This was done by simulating the full geometry of the CC, as well as a perovskite thin film with varying σ_{dark} located in it. The σ_{dark} of the perovskite thin film was varied computationally, and simultaneously the response of the cavity, i.e. the resulting frequency scan, to the varying σ_{dark} was also computed. Hence, the corresponding R_0 was obtained. In this manner, the calibration curves relating R_0 and σ_{dark} were constructed (see **Figure 2b**).[5] These enabled the precise quantitative research for perovskite thin films with varying σ_{dark}.

It is noteworthy that the accuracy in the determination of σ_{dark} from R_0 depend on the lower and upper detection limits of the SSMC technique.
The lower limit is determined by the intrinsic conductivity of quartz, an electrical insulator layer on which the perovskite thin films are deposited. Quartz substrates may present different properties, due to factors such as the residual water content in them, which affects the conductivity and relative permittivity, or different thickness. Hence, the quartz substrates can affect the determination of σ_{dark} of the perovskite films. This effect is particularly relevant when R_0 of the perovskite film is close to the lower detection limit, *i.e.* when the frequency scans of the perovskite thin film and quartz are almost the same ($R_{0,perovskite} \approx R_{0,quartz}$). For this reason, in particular for the 0 days aged SnI_2 precursor series, we took the effect of the quartz substrate into account to determine accurately σ_{dark} of the perovskite layers. We measured the frequency scans of specific quartz substrates and of the perovskite films deposited on them. We first modelled the resonant frequency dips of each quartz substrate to determine the conductivity properties. Then, when modelling the conductive perovskite thin film deposited on it, we corrected for the specific quartz substrate properties. In this way, we attributed the dip deepening in the frequency scan mainly to the microwave absorption due to conductivity of the perovskite film. This allowed us to estimate σ_{dark} with even higher accuracy.
Moreover, we attributed the microwave absorption and the correspondent dip deepening of the perovskite film with respect to the quartz substrate exclusively to free, mobile carriers absorption (doping). Yet, any other process involving charges moving in the dielectric perovskite (*e.g.* dipoles rotations, ions displacements) can cause microwave absorption, although the effect of these processes is supposed to be limited. Nevertheless, this means that σ_{dark} can be slightly overestimated.
On the other hand, the upper limit depends on the magnitude of the microwave perturbation, which in case of films with very high σ_{dark} can become very strong and affect the determination of σ_{dark}. Indeed, when R_0 was close to the upper detection limit, as for the perovskite layer with 0 mol% SnF_2 in **Figure 3c** in the main text, σ_{dark} was estimated with lower accuracy (see **Figure 3d** in the main text).

M 2) SIMsalabim, 1D drift-diffusion simulator

The time- and laser light-dependent TRMC traces can be fitted with SIMsalabim, an open-source 1D drift-diffusion simulator for semiconductor materials that takes into account the photogeneration of both electrons and holes, their recombination and trapping, the effect of ions and dopants, and self-consistently solves the electric field that results from all charged species.[6]

In the drift-diffusion model, the coupled set of continuity equations with the Poisson equation is solved. The Poisson equation in **Equation (S9)** relates the potential to the charge carrier distribution.

$$\frac{\partial}{\partial x}\left(\varepsilon(x)\frac{\partial V(x)}{\partial x}\right) = -e(p(x) - n(x) + C(x)) \qquad (9)$$

Where x is the position in the system, ε is the dielectric constant, V is the potential, e is the elementary charge, n and p are respectively the electron and hole density, and C is the sum of all additional charges, like ionized traps or ions.

The time-dependent continuity equations for electrons and holes are shown in **Equations (S10) and (S11)**.

$$\frac{\partial n(x)}{\partial t} - \frac{1}{e}\frac{\partial J_n(x)}{\partial x} = G(x) - R(x) \qquad (10)$$

$$\frac{\partial p(x)}{\partial t} - \frac{1}{e}\frac{\partial J_p(x)}{\partial x} = G(x) - R(x) \qquad (11)$$

Where J_n and J_p are respectively the electron and hole current density, G is the generation rate and R is the recombination rate.

J_n and J_p can be written in terms of a drift and diffusion components, as shown in **Equations (S12) and (S13)**.

$$J_n = -en(x)\mu_n(x)\frac{\partial V(x)}{\partial x} + eD_n\frac{\partial n(x)}{\partial x} \qquad (12)$$

$$J_p = -ep(x)\mu_p(x)\frac{\partial V(x)}{\partial x} + eD_p\frac{\partial p(x)}{\partial x} \qquad (13)$$

Where μ_n and μ_h are the charge carrier mobilities and D_n and D_h are the carrier diffusion coefficients. Discretized and coupled with suitable boundary conditions, these equations are numerically solved in an iterative algorithm.

To simulate a TRMC measurement with SIMsalabim, the perovskite thin films were defined in terms of the model parameters. Besides, an accurate representation of the laser pulse, including the instrumental response function, was created to obtain a time-dependent generation profile. The different laser intensities were also taken into account. Then, the TRMC signal (trace) is calculated from the output by using **Equation (S7)**.

To fit the simulated TRMC traces with the experimental TRMC traces, an automated fitting procedure was used with the aim to minimize the fit error, which is quantified as the normalized area between the simulated and the experimental TRMC signals.

Table S1: Location above the valence band edge of shallow (bulk) trap states and deep (surface) trap states within the bandgap, obtained as fitted parameters of the 1D drift-diffusion modelling of the TRMC traces of perovskite thin films with varying SnF_2 concentrations.

mol% SnF_2	Shallow (bulk) trap states (eV)	Deep (surface) trap states (eV)
0	1.13	0.62
1	1.11	0.66
2	1.06	0.66
5	1.06	0.66
10	1.06	0.66
20	1.06	0.66

Calculations (C)

C 1) Lattice parameter

The lattice parameter, *a*, of the pseudo-cubic perovskite crystal structure was obtained by applying **Equation (S14)**,[7] in reference to the XRD pattern of $Cs_{0.25}FA_{0.75}Sn_{0.5}Pb_{0.5}I_3$ perovskite thin films with varying SnF_2 content in **Figure S10**.

$$a = \frac{\lambda}{2 \cdot sin\theta}\sqrt{(h^2 + k^2 + l^2)} \tag{14}$$

Where *sin θ* is the sine of half the diffraction angle 2*θ* at which a perovskite XRD peak with Miller indexes (hkl) is located. The wavelength of the Cu-K_α X-rays used in these measurements λ = 1.54056 Å.

C 2) Molar extinction coefficient of SnI_4 in toluene

To calculate the molar extinction coefficient of SnI_4 in toluene, ε_{SnI_4}, we first measured the absorbance spectra of reference SnI_4 dissolved in toluene at different concentrations, shown in **Figure S4a**. Then, we calculated ε_{SnI_4} according to **Equation (S15)**.

$$O.D.(\lambda = 365\ nm) = \varepsilon_{SnI_4} l[SnI_4] \tag{15}$$

Where *O.D.* is the absorbance of SnI_4 at the main absorption peak at λ ~ 365 nm, l the optical path in solution of 0.20 cm (see **SI – E/M 5**) and [SnI_4] the known concentration of SnI_4. In **Figure S4b**, we plotted *O.D.* (λ ~ 365 nm) as a function of [SnI_4]. We fitted such data with a line, whose slope divided by the optical path length in the solution is equal to ε_{SnI_4}.

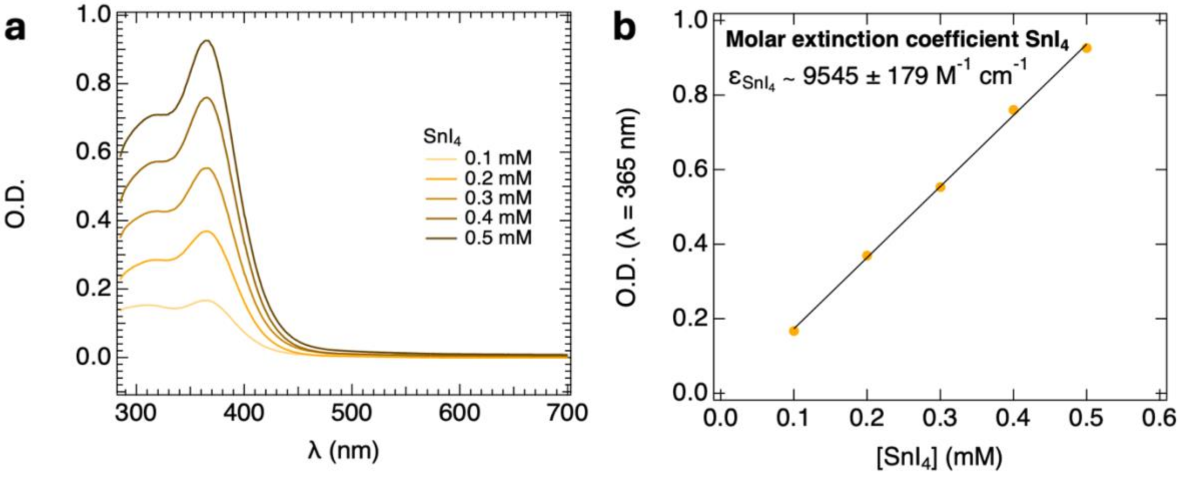

Figure S4: (a) Absorbance in solution of SnI_4 dissolved at different concentrations in toluene. The main absorption peak is at λ ~ 365 nm. (b) Molar extinction coefficient of SnI_4 in toluene, ε_{SnI_4}, calculated from the data in (a).

C 3) Linking the fraction of Sn^{4+} to Sn^{2+} in solution to the dark free holes concentration p_0 in the perovskite film (*i.e.* to the fraction of Sn^{4+} to Sn^{2+} in the crystal)

First, we calculated the initial concentration of Sn^{4+} with respect to Sn^{2+} in solution. Similarly to **Figure 1** and **Table 1** in the main text, we analyzed by absorption spectroscopy a toluene solution obtained by washing slightly aged SnI_2 precursor, stirring overnight and filtering, as shown in **Figure S5a**. In this way, we extracted any formed SnI_4. By comparing it with a reference spectrum of SnI_4 dissolved in toluene and knowing ε_{SnI_4} from **SI – C2**, we calculated a fraction Sn^{4+} to Sn^{2+} in solution of 0.013%, as indicated in **Table S2**.

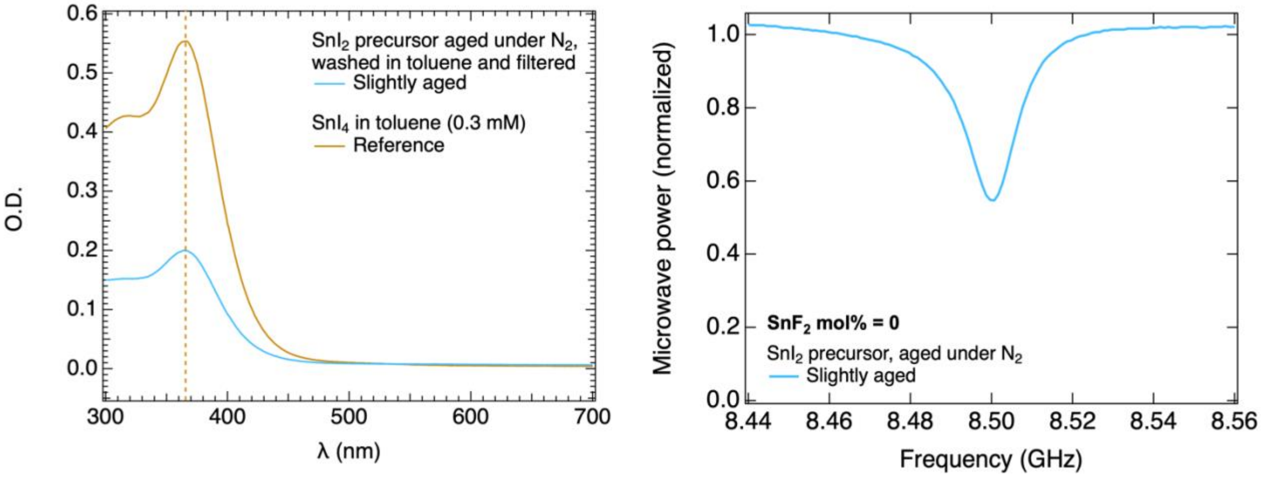

Figure S5: (a) Absorbance spectrum of a toluene solution obtained by aging SnI_2 precursor in a glovebox, extraction and filtering. The spectrum, recorded in a 0.20 cm-thick cuvette, is compared to the reference absorption spectrum of SnI_4 dissolved in toluene (in yellow). (b) SSMC frequency scan of a perovskite layer with 0 mol% SnF_2 prepared by using the same SnI_2 precursor in (a).

Table S2: Concentration of SnI_4 in toluene solution, obtained by washing in 1.0 mL of toluene and filtering of ~ 289 mg of slightly aged SnI_2 precursor, and fraction of oxidized Sn^{4+}.

Washed SnI_2	$[\mathbf{SnI_4}]$ (mM)	Fraction Sn^{4+} to Sn^{2+} (%)
Slightly aged	0.10	0.013

Then, we calculated the concentration of Sn^{4+} with respect to Sn^{2+} in the crystallized perovskite film without added SnF_2. For this, we prepared the perovskite layer with the same slightly aged SnI_2 precursor used for the absorption measurement in **Figure S5a** and we measured the SSMC frequency scan of the layer, shown in **Figure S5b**. From this, we obtained σ_{dark} by using the calibration curve in **Figure 2b** in the main text and we calculated the concentration of dark free holes p_0 amounting to 6.6 x 10^{16} cm^{-3}. Since the concentration of Sn^{4+} in the crystallized perovskite film, $c_{Sn^{4+}}$, is associated with half of the dark free holes concentration p_0, we calculated $c_{Sn^{4+}}$ as shown in **Equation (S16)**.

$$c_{Sn^{4+}} = \frac{p_0}{2} = \frac{6.6 \cdot 10^{16}}{2} = 3.3 \cdot 10^{16}\ cm^{-3} \tag{16}$$

At this point, we compared $c_{Sn^{4+}}$ in the perovskite film without added SnF_2 to the concentration of Sn^{2+} in the ideally perfect (not oxidized) perovskite crystal structure. The density of Sn^{2+} ions in the ideally perfect perovskite crystal is calculated in **Equation (S17)**.

$$\rho_{Sn^{2+}} = \frac{m_{Sn^{2+}}}{V_{cell}} = \frac{\frac{0.5 \cdot P_{A,Sn^{2+}}}{N_A}}{a^3} = 0.399\ g\ cm^{-3} \tag{17}$$

Where the atomic weight of tin is $P_{A,Sn2+}$ = 118.71 g mol^{-1}, N_A is the Avogadro's number (N_A = 6.022 x 10^{23} mol^{-1}) and V_{cell} is the volume of the unit cell, calculated by assuming a pseudo-cubic perovskite crystal structure and using the value of *a* obtained by **Equation (S14)** and shown in **Figure S10c**.

It follows that the concentration of Sn^{2+} in the ideally perfect perovskite crystal, $c_{Sn^{2+}}$, can be obtained by **Equation (S18)**.

$$c_{Sn^{2+}} = \frac{\rho_{Sn^{2+}} \cdot N_A}{P_{A,Sn^{2+}}} \sim 2\ x\ 10^{21}\ \ cm^{-3} \tag{18}$$

By comparing $c_{Sn^{4+}}$ to $c_{Sn^{2+}}$, we calculated that ~ 0.0016% of the Sn^{2+} in the crystallized perovskite film without added SnF_2 is involved in doping.

Hence, we compared the fraction of Sn^{4+} with respect to Sn^{2+} in the perovskite precursor solution (0.013%) to the fraction of Sn^{4+} with respect to Sn^{2+} in the ideally perfect perovskite film without added SnF_2 (~ 0.0016%). From this, we calculated that ~ 12% of the Sn^{4+} in the perovskite solution leads to doping in the perovskite layer.

C 4) Fraction of Sn^{4+} to Sn^{2+} in the crystallized perovskite films without SnF_2, made with differently aged SnI_2 precursors

For the depositions made with differently aged SnI_2 precursors in **Figure 3** in the main text, we calculated p_0 for the perovskite films without added SnF_2. From this, we derived the corresponding $c_{Sn^{4+}}$ in these films, given that p_0 derives from half of $c_{Sn^{4+}}$. Moreover, by comparing $c_{Sn^{4+}}$ to the $c_{Sn^{2+}}$ in **Equation (S18)**, we calculated which fraction of the ideally perfect crystallized perovskite film without added SnF_2 is oxidized to Sn^{4+}. The results are shown in **Table S3**.

Table S3: Values of p_0 and corresponding $c_{Sn^{4+}}$ for the perovskite films without added SnF_2 belonging to the depositions made with differently aged SnI_2 precursors in **Figure 3** in the main text, and fraction of oxidized Sn^{4+} in the crystallized film.

Aged SnI_2 precursor	p_0 (cm^{-3})	$c_{Sn^{4+}}$ (cm^{-3})	Fraction Sn^{4+} to Sn^{2+} (%)
0 days	7.1 x 10^{16}	3.5 x 10^{16}	0.0018
2 days	1.6 x 10^{17}	8.1 x 10^{16}	0.0040
20 days	4.1 x 10^{17}	2.0 x 10^{17}	0.0102

As derived in **SI – C3**, we know that the fraction Sn^{4+} to Sn^{2+} in the crystallized film corresponds to ~ 12% of the initial Sn^{4+} in the perovskite solution. By comparing these fractions, we can calculate the initial concentration of Sn^{4+} (in the form of SnI_4) and which fraction of SnI_2 in the perovskite precursor solution is

oxidized to Sn^{4+} (in the form of SnI_4) for the depositions made with differently aged SnI_2 precursors in **Figure 3** in the main text. The results are shown in **Table 3** in the main text.

C 5) Excess concentration of SnF_2 required to suppress doping in the perovskite film

The molarity, *M*, of the $Cs_{0.25}FA_{0.75}Sn_{0.5}Pb_{0.5}I_3$ perovskite precursor solution (*M* = 1.55 M, as described in **SI – E/M 2**) is expressed in **Equation (S19)** considering 1 mL of solution.

$$M = \frac{0.25 \cdot n_{CsI} + 0.75 \cdot n_{FAI} + 0.5 \cdot n_{SnI_2} + 0.5 \cdot n_{PbI_2}}{1} = 1.55 \cdot 10^{-3}\, M \qquad (19)$$

This means that ¼ of the moles in solution are of SnI_2 (corresponding to the expected concentration of Sn^{2+}), as shown in **Equation (S20)**.

$$n_{Sn^{2+}(SnI_2)} = \frac{M}{4} = \frac{1.55 \cdot 1 \cdot 10^{-3}}{4} = 3.9 \cdot 10^{-4}\, mol \qquad (20)$$

We considered the oxidation of 0.015% of the 0 days aged SnI_2 precursor, as derived in **SI – C4** and shown in **Table 3** in the main text. From this, we calculated how many moles of Sn^{2+} are already oxidized to Sn^{4+} in the perovskite precursor solution without SnF_2 **Equation (S21)**.

$$n_{Sn^{4+}(SnI_4)} = 0.00015 \cdot n_{Sn^{2+}(SnI_2)} = 0.00015 \cdot 3.9 \cdot 10^{-4}\, mol = 5.8 \cdot 10^{-8}\, mol \qquad (21)$$

This means that 5.8 x 10^{-8} mol out of the expected 3.9 x 10^{-4} mol of ideally pure 0 days aged SnI_2 precursor are oxidized to SnI_4 in the perovskite precursor solution without SnF_2.

Then, we calculated SnF_2 added in 1 mL solution, n_{SnF_2}, which is a mol% of the ideally present $n_{Sn^{2+}(SnI_2)}$. We compared n_{SnF_2} to $n_{Sn^{4+}(SnI_4)}$ in solution in **Table S4**. In this way, we studied the excess of SnF_2 (for varying SnF_2 additions) over Sn^{4+} initially present in the perovskite precursor solution without SnF_2.

Table S4: Excess of SnF_2 over the Sn^{4+} initially present in the 0 days aged SnI_2 perovskite precursor solution without SnF_2.

mol% SnF_2 w.r.t. SnI_2	n_{SnF_2} (mol)	Excess of SnF_2
0	0	0
1	3.9 x 10^{-6}	+ 69
2	7.8 x 10^{-6}	+ 137
5	1.9 x 10^{-5}	+ 343
10	3.9 x 10^{-5}	+ 687
20	7.8 x 10^{-5}	+ 1374

Steady State Microwave Conductance (SSMC) – Dark conductivity and doping & Time-resolved Microwave Conductivity (TRMC) – Photogenerated charge carrier dynamics

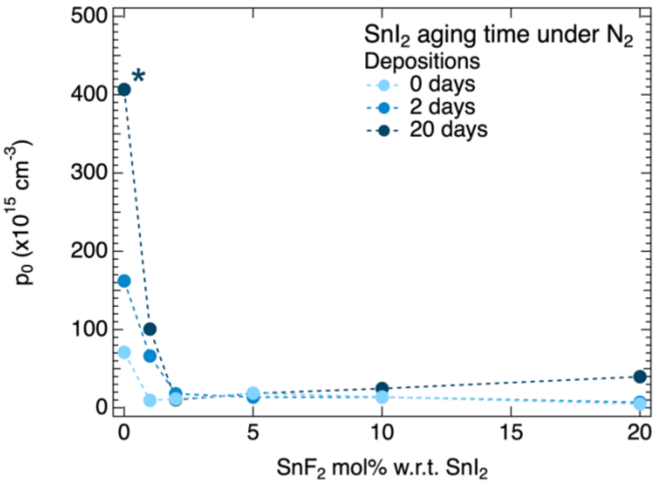

Figure S6: Effect on p_0 of using SnI_2 precursor of different purity, i.e. aged for different times in a glovebox, and of varying SnF_2 concentrations in $Cs_{0.25}FA_{0.75}Sn_{0.5}Pb_{0.5}I_3$ perovskite thin films. The relative samples belong to the depositions shown in **Figures 3a**, **3b and 3c** in the main text. The marker (*) next to a data point indicates lower accuracy in the determination of p_0, as the resonant dip for the corresponding layer is close to the upper detection limit of the SSMC technique.

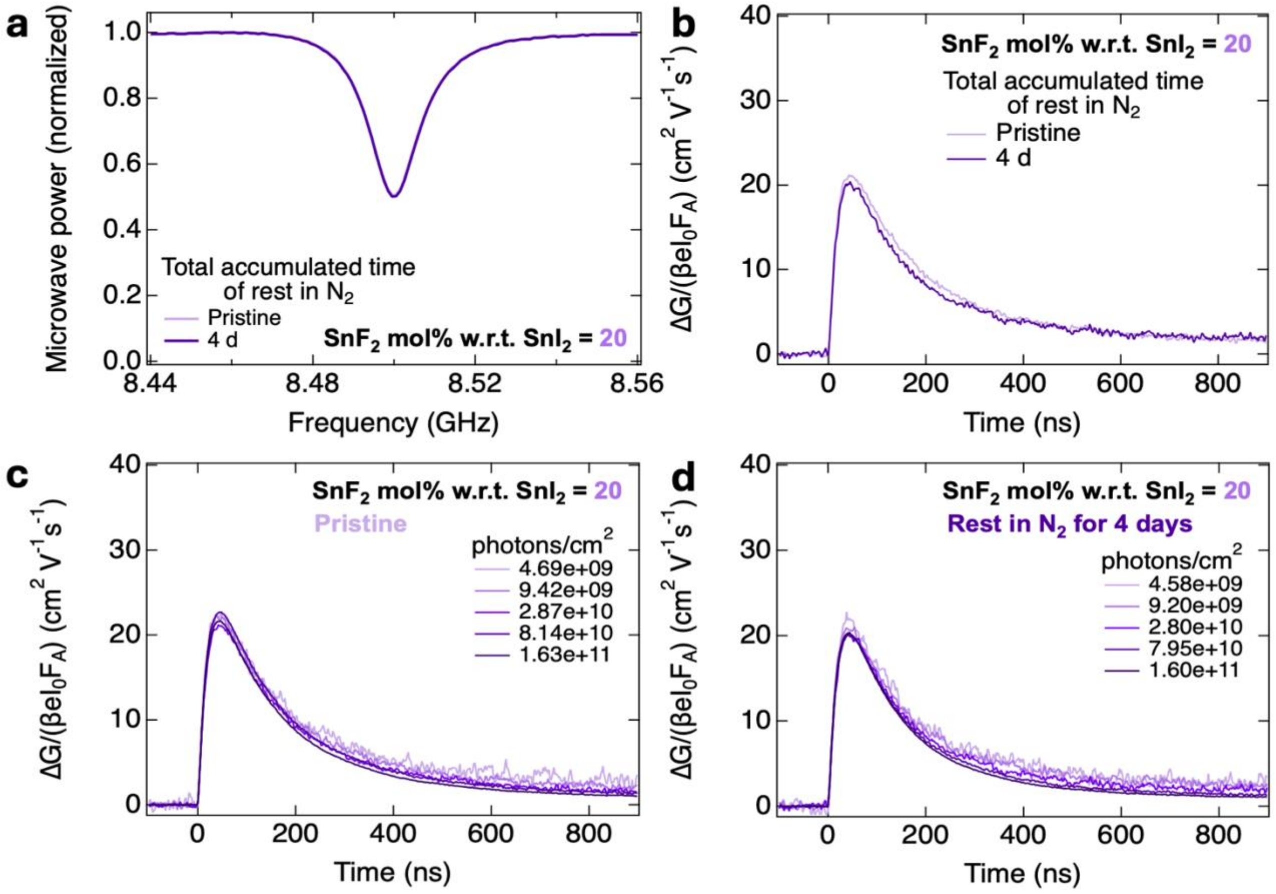

Figure S7: Comparison of SSMC measurements and TRMC measurements showing the effect of resting in N_2 on a perovskite film with 20 mol% SnF_2. (a) SSMC measurements and (b) TRMC measurements comparing the layer in pristine conditions (light purple) and after resting in N_2 for ~ 4 days (in dark purple). The TRMC measurements in (b) were performed at the same laser intensity (~ 2-3 x 10^{10} photons cm^{-2}) and same excitation wavelength (λ = 800 nm). Intensity-dependent TRMC measurements for the perovskite film in (c) pristine conditions and (d) after resting in N_2 for ~ 4 d. The TRMC traces in (c)(d) were measured with different laser intensities and at the same excitation wavelength (λ = 800 nm). All the TRMC traces are corrected for the absorbed fraction of light in **Figure S9** at the excitation wavelength *λ* = 800 nm.

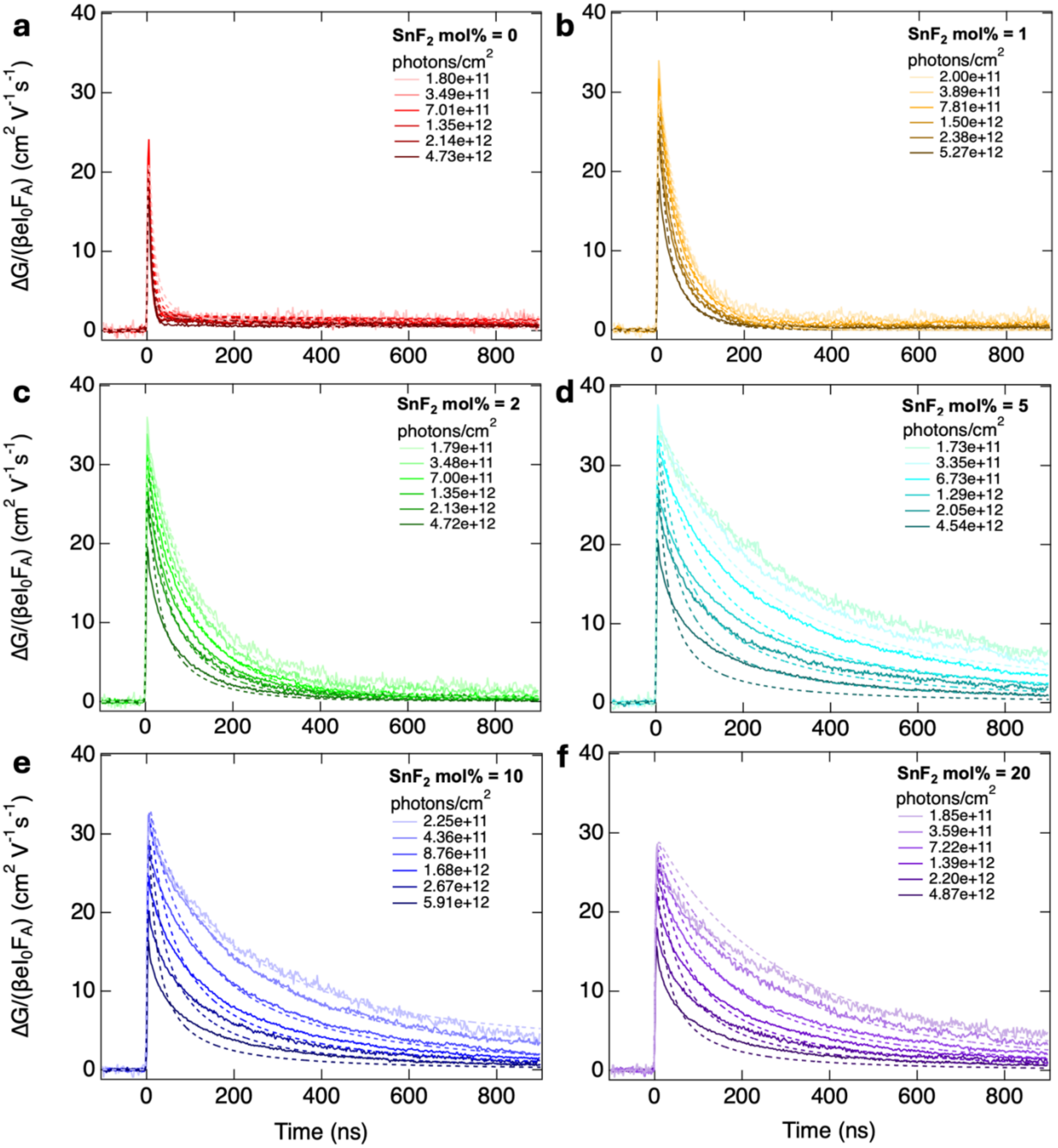

Figure S8: TRMC traces of $Cs_{0.25}FA_{0.75}Sn_{0.5}Pb_{0.5}I_3$ perovskite thin films with varying SnF_2 concentrations. All samples belong to the best-performing deposition in **Figure 3a** in the main text, produced with 0 days aged SnI_2 precursor. The solid lines represent the experimental time- and laser-dependent TRMC traces obtained by using a microwave OC, while the dashed lines correspond to the modelled TRMC traces resulting from the 1D drift-diffusion simulator. All the TRMC traces are corrected for the absorbed fraction of light in **Figure S9** at the excitation wavelength λ = 800 nm.

UV-Vis-NIR Spectroscopy (UV-Vis) – Absorption coefficient and bandgap energy

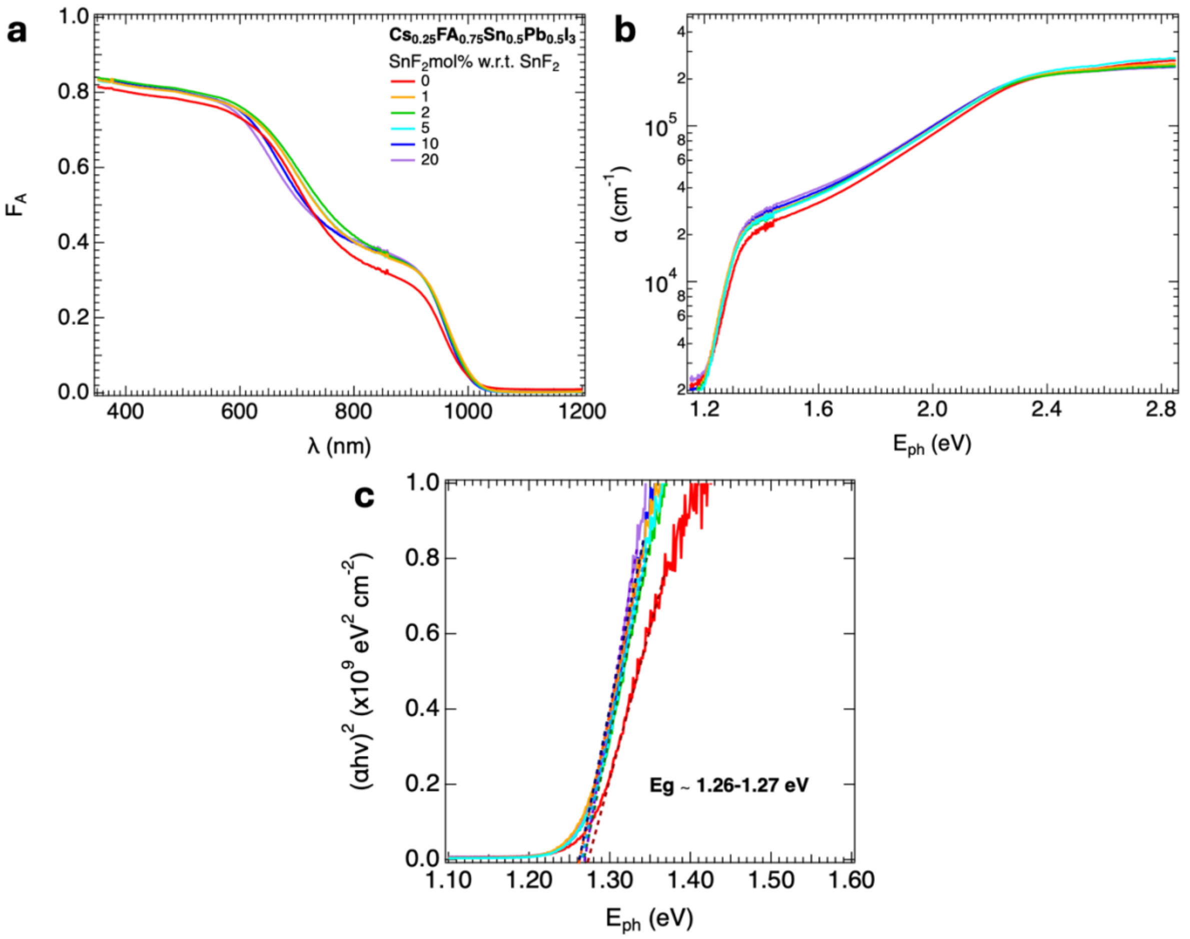

Figure S9: Optical properties of $Cs_{0.25}FA_{0.75}Sn_{0.5}Pb_{0.5}I_3$ perovskite thin films with varying SnF_2 concentrations. All samples belong to the best-performing deposition in **Figure 3a** in the main text, produced with 0 days aged SnI_2 precursor. (a) Absorptance spectra, (b) absorption coefficient spectra measured by UV-Vis and (c) derived Tauc plots showing the bandgap energy of perovskite thin films with varying SnF_2 concentrations.

X-Ray Diffraction (XRD) – Crystal structure

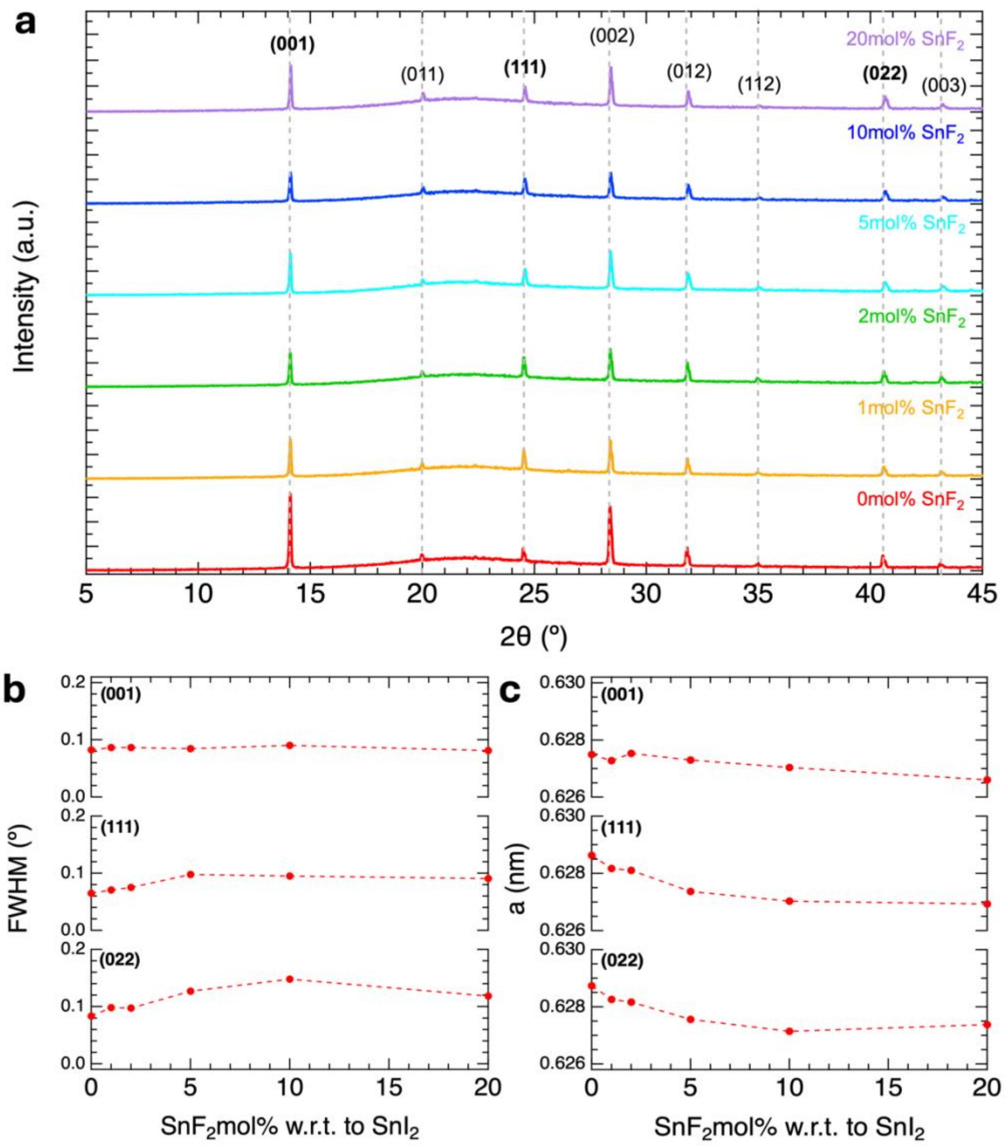

Figure S10: Crystal structure properties of $Cs_{0.25}FA_{0.75}Sn_{0.5}Pb_{0.5}I_3$ perovskite thin films with varying SnF_2 concentrations. All samples belong to the best-performing deposition in **Figure 3a** in the main text, produced with 0 days aged SnI_2 precursor. (a) XRD patterns showing the Miller indexes belonging to the characteristic diffraction peaks of the pseudocubic crystal phase of perovskite with no preferential crystal orientation, in line with the literature.[8-10] (b) Full-width half-maximum (FWHM). The FWHM is an indicator of disorder, such as grain boundaries and residual strains in the crystal. We observed a similar crystallinity for all samples regardless of SnF_2 concentration. (c) Crystal lattice parameter, *a*, of the three perovskite peaks corresponding to different sets of crystal planes (001) at $2\theta \simeq 14.0°$, (111) at $2\theta \simeq 24.5°$, and (022) $2\theta \simeq 40.5°$ (see **SI – C 1** for the detailed calculations). We observed minimum variations in *a* as a function of the SnF_2 concentration.

X-ray Photoelectron Spectroscopy (XPS) – Elemental composition and depth profiling

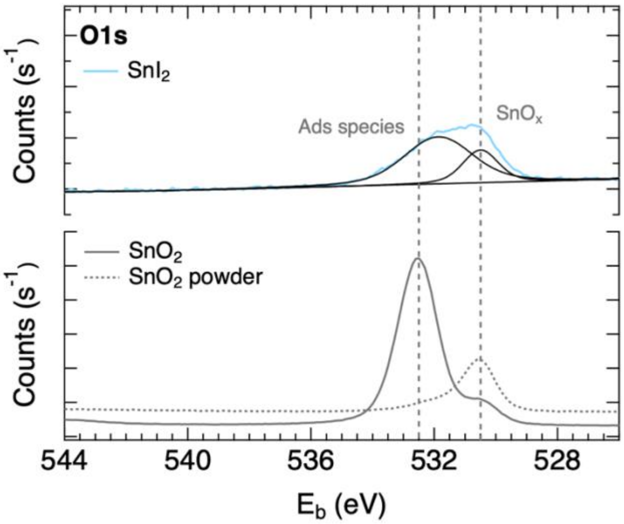

Figure S11: XPS analysis showing the O1s core levels peaks of 0 days aged SnI_2 precursor and reference SnO_2, in the form of thin film (solid line) and powder (dotted line). The fitted XPS peak located at E_b ~ 531.9 eV and defined as *Ads species* is likely a collection of narrower XPS peaks corresponding to O-containing adsorbed species, *i.e.* O-H, O=C and O-C species (going from low to high E_b) as reported.[11]

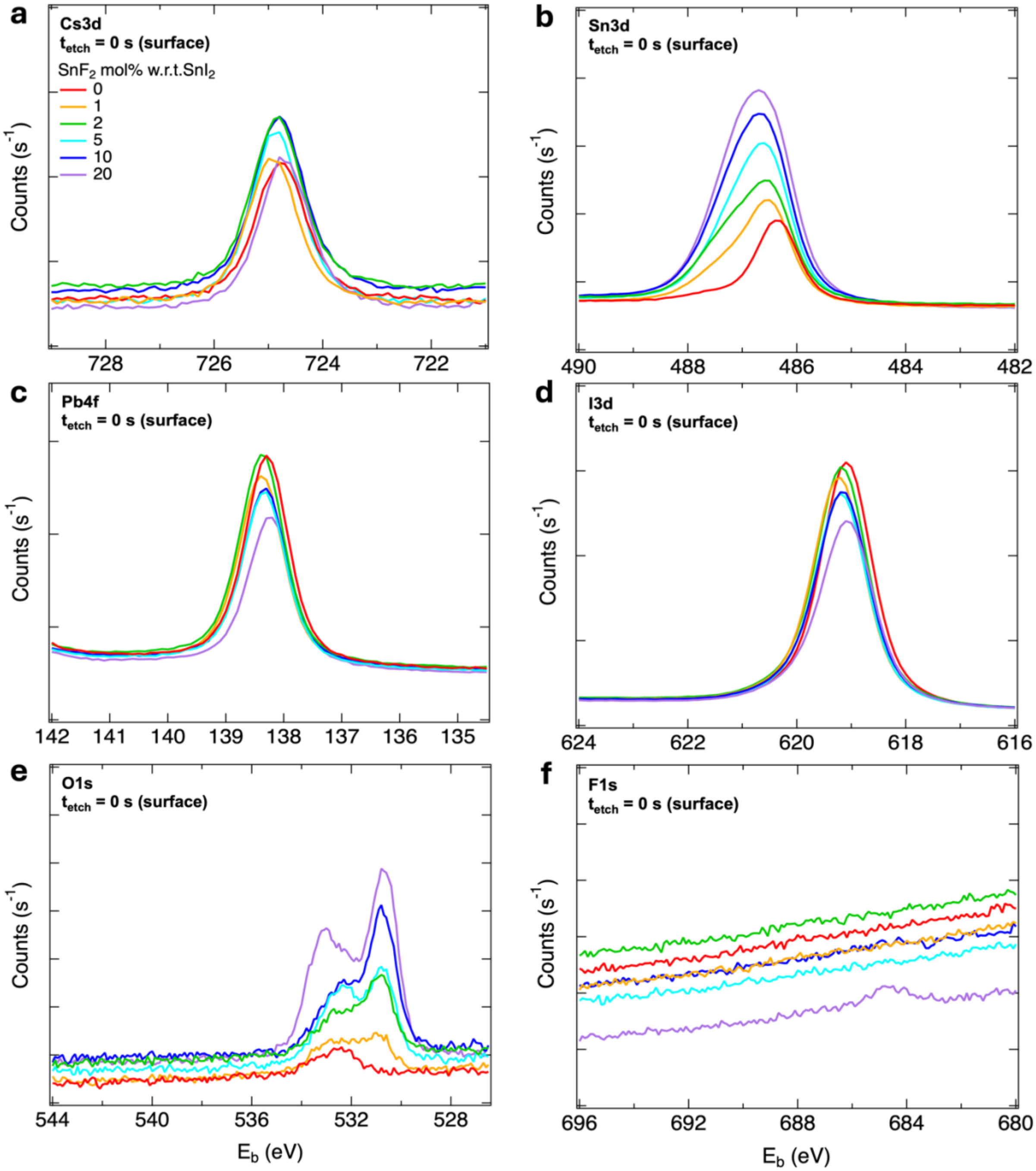

Figure S12: XPS surface analysis showing the (a) Cs3d, (b) Sn3d, (c) Pb4f, (d) I3d, (e) O1s, (f) F1s, and core levels peaks of $Cs_{0.25}FA_{0.75}Sn_{0.5}Pb_{0.5}I_3$ perovskite thin films with varying SnF_2 concentration. All samples belong to the best-performing deposition in **Figure 3a** in the main text, produced with 0 days aged SnI_2 precursor. The intensity of the XPS signal for the different electron transitions and elements orbitals is shown as a function of the electron binding energy, E_b.

a

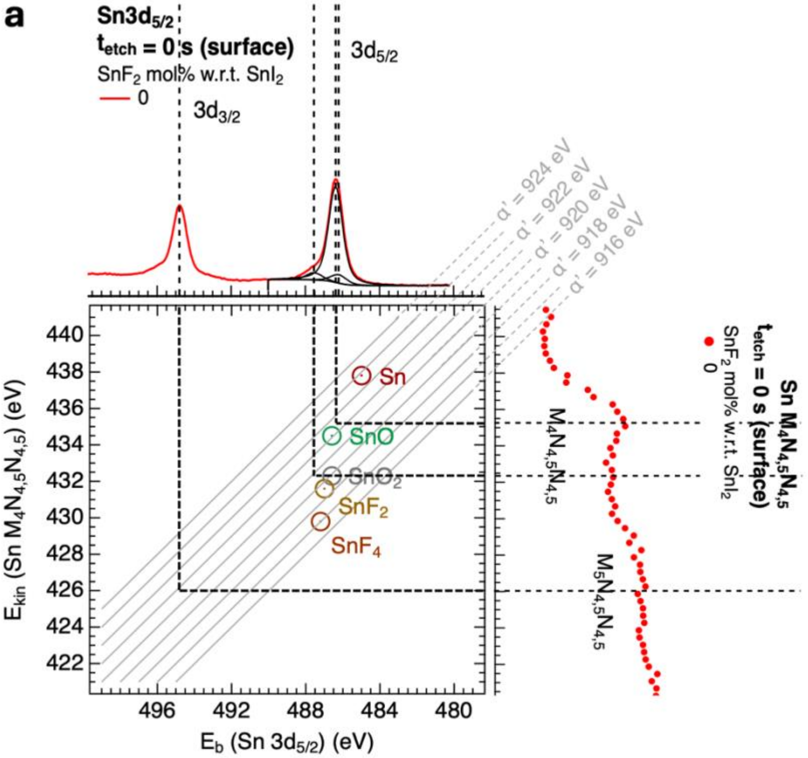

Sn3d5/2
tetch = 0 s (surface)
SnF2 mol% w.r.t. SnI2
0
3d3/2
3d5/2
α' = 924 eV
α' = 922 eV
α' = 920 eV
α' = 918 eV
α' = 916 eV
Sn
SnO
SnO2
SnF2
SnF4
M4N4,5N4,5
M5N4,5N4,5
Sn M4N4,5N4,5
tetch = 0 s (surface)
SnF2 mol% w.r.t. SnI2
0
Ekin (Sn M4N4,5N4,5) (eV)
Eb (Sn 3d5/2) (eV)

b

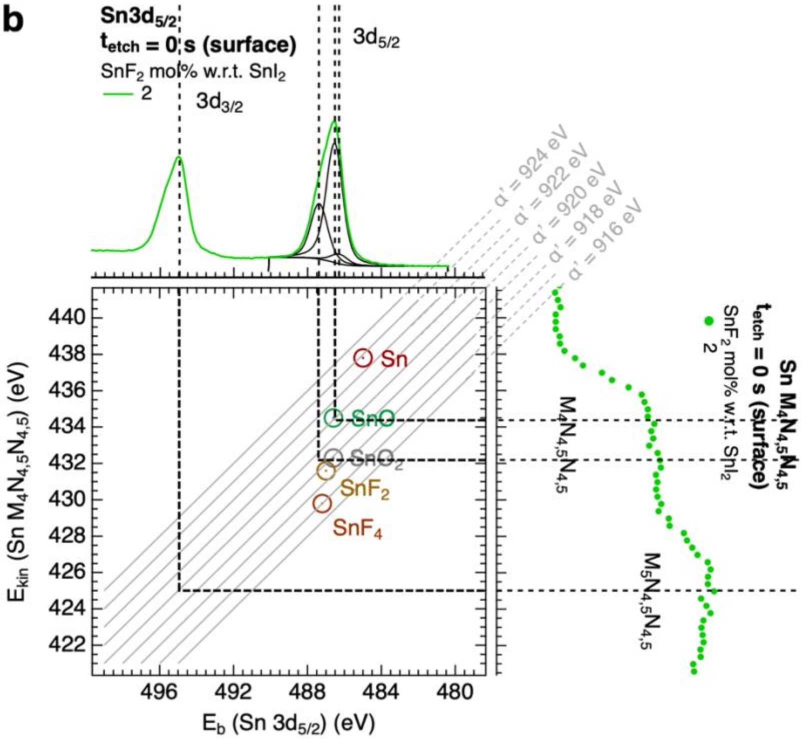

Sn3d5/2
tetch = 0 s (surface)
SnF2 mol% w.r.t. SnI2
2
3d3/2
3d5/2
α' = 924 eV
α' = 922 eV
α' = 920 eV
α' = 918 eV
α' = 916 eV
Sn
SnO
SnO2
SnF2
SnF4
M4N4,5N4,5
M5N4,5N4,5
Sn M4N4,5N4,5
tetch = 0 s (surface)
SnF2 mol% w.r.t. SnI2
2
Ekin (Sn M4N4,5N4,5) (eV)
Eb (Sn 3d5/2) (eV)

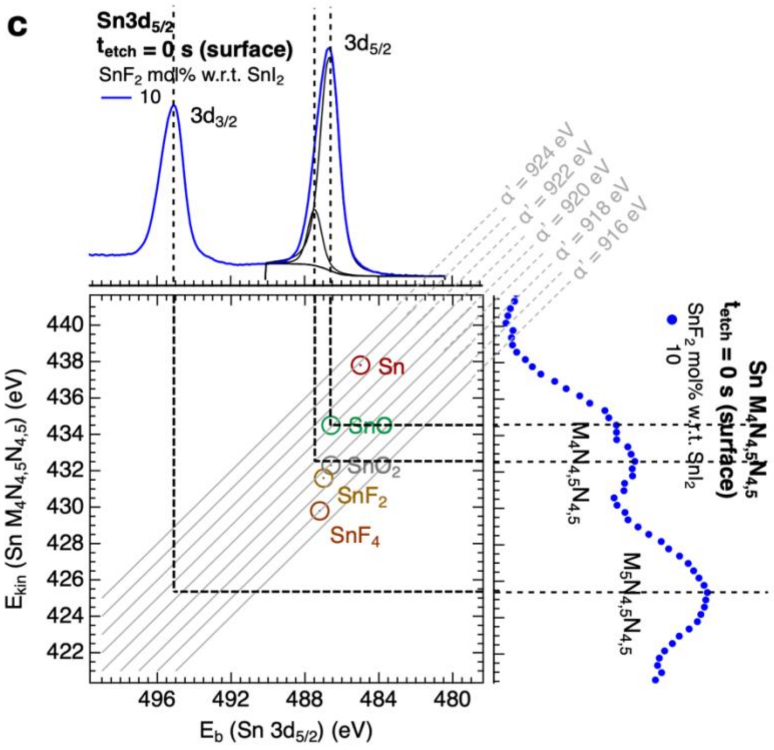

Figure S13: Wagner plots derived from the surface XPS analysis of Sn for Sn-Pb perovskite films with (a) 0, (b) 2 and (c) 10 mol% SnF_2 addition. Following the method reported in the literature,[12] the maxima of the peaks around the Sn $3d_{5/2}$ (core-level electrons) and around the Sn $M_4N_{4,5}N_{4,5}$ and $M_5N_{4,5}N_{4,5}$ (Auger electrons) features construct points on the Wagner plot. We retrieved the Auger emission spectrum *vs* kinetic energy (E_{kin}) from the main surface XPS surveys *vs* binding energy (E_b) of the Sn-Pb perovskite films. The references for the Sn and SnO species are obtained from the literature (respectively, in dark red and green),[12] while the references for SnO_2, SnF_2, and SnF_4 are obtained from spin-coated reference layers (respectively, in grey, dark yellow and brown). The XPS analysis for the SnF_2 and SnF_4 reference layers is shown in **Figure S14** and **Table S5**. More information about the reference layers synthesis can be found in the **SI – E/M 3**. The references points are compared to the Sn-Pb perovskites films points to identify the chemical species generating the XPS peaks in **Figure S15** for the Sn-Pb perovskite films. The diagonal lines (in light grey) correspond to constant values of modified Auger parameter, *α'*, equal to the sum of E_{kin} and E_b, which are also used for comparisons with literature references. We underline that the spectral resolution, the limited information in the literature about XPS on Sn-containing perovskites and the not exact match with other reported values hindered a confident identification of all the XPS peaks originated by Auger and core-level electrons. Hence, the Wagner plots analysis is just indicative of the perovskite and SnO_x species in the layers.

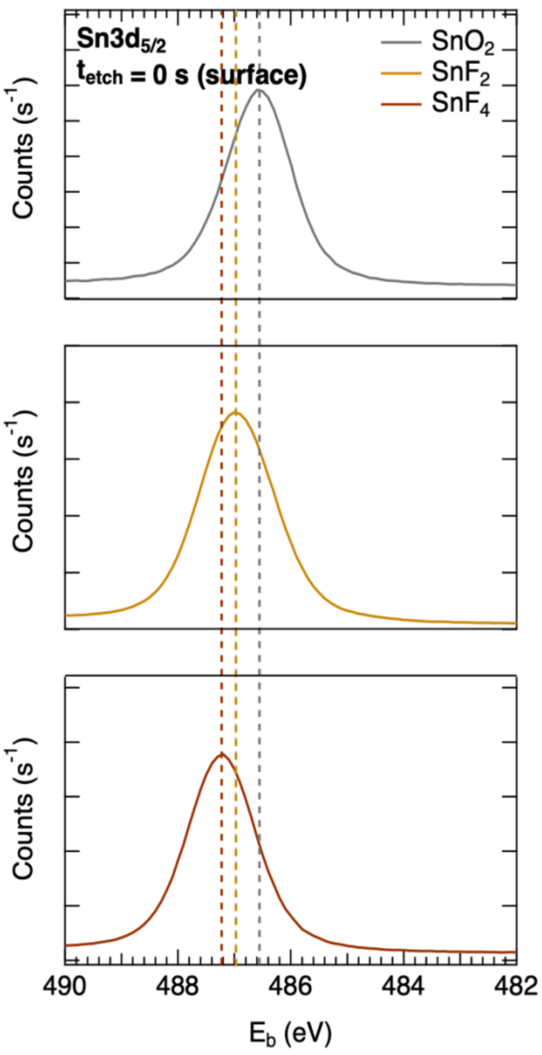

Figure S14: XPS surface analysis showing the Sn3d core levels peaks of spin-coated SnO_2, SnF_2, and SnF_4 reference layers (respectively, in grey, dark yellow and brown).

Table S5: Kinetic energy, E_{kin}, binding energy, E_b, and modified Auger parameters, *α'*, for the Sn3d core levels surface XPS peaks of spin-coated SnO_2, SnF_2, and SnF_4 reference layers. To obtain E_{kin}, we retrieved the Auger emission spectrum *vs* E_{kin} from the main surface XPS surveys *vs* E_b in **Figure S14**, in the same way as **Figure S13**.

**Sn3d surface XPS**	E_{kin}	E_b	*α'*
SnO_2	~ 432.3	~ 486.6	~ 918.9
SnF_2	~ 431.6	~ 487.0	~ 918.6
SnF_4	~ 429.8	~ 487.2	~ 917.0

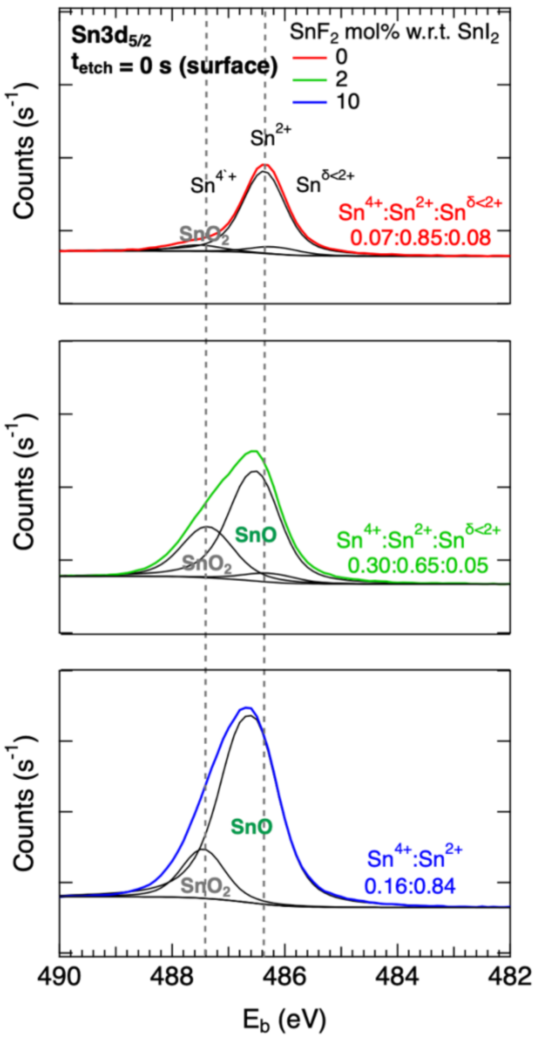

Figure S15: XPS surface analysis and peak fitting showing the Sn3d core levels peaks of $Cs_{0.25}FA_{0.75}Sn_{0.5}Pb_{0.5}I_3$ perovskite thin films with 0 (in red), 2 (in green) and 10 (in blue) mol% SnF_2 additions. All samples belong to the deposition in **Figure 3a** of the main text, produced with 0 days aged SnI_2 precursor. The intensity of the XPS signal for the different electron transitions and elements orbitals is shown as a function of the electron binding energy, E_b. The chemical state analysis of this surface XPS scan is performed prior to any etching to avoid damage by the Ar^+ sputter gun. The results from peak fitting are also shown (solid lines in black). These were attributed to different oxidation species, whose ratio for each film is indicated as Sn^{4+}:Sn^{2+}:$Sn^{\delta<2+}$. From the Wagner plots for the surface XPS analysis of Sn in **Figure S13**, for the sample with 0 mol% we attributed the main XPS fitted peak at E_b ~ 486.4 eV to Sn^{2+} in the perovskite crystal structure. For the film with 2 SnF_2, we attributed the main fitted peak at E_b ~ 486.5 eV to Sn^{2+} in the form of SnO. The fitted peak at the highest E_b ~ 487.4 eV appearing upon SnF_2 addition is attributed to Sn^{4+} in the form of SnO_2 on the surface. This seems also the case for the film with 10 mol% SnF_2, presenting two fitted peaks at E_b ~ 486.6 eV and E_b ~ 487.5 eV, respectively.

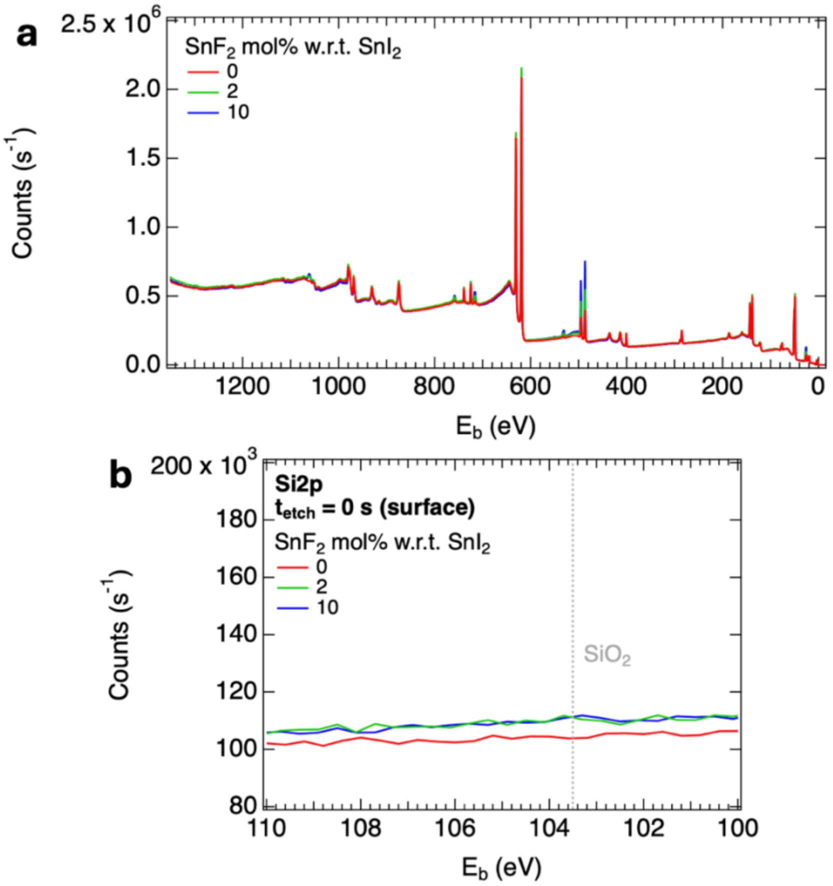

Figure S16: Surface (a) XPS survey and (b) zoom on the surface XPS scan of the Si2p orbitals, showing the reference SiO_2 reported in the literature.[13] This demonstrates that there were no contributions to the surface XPS scans of the Sn-Pb perovskite films from the underlying quartz substrates.

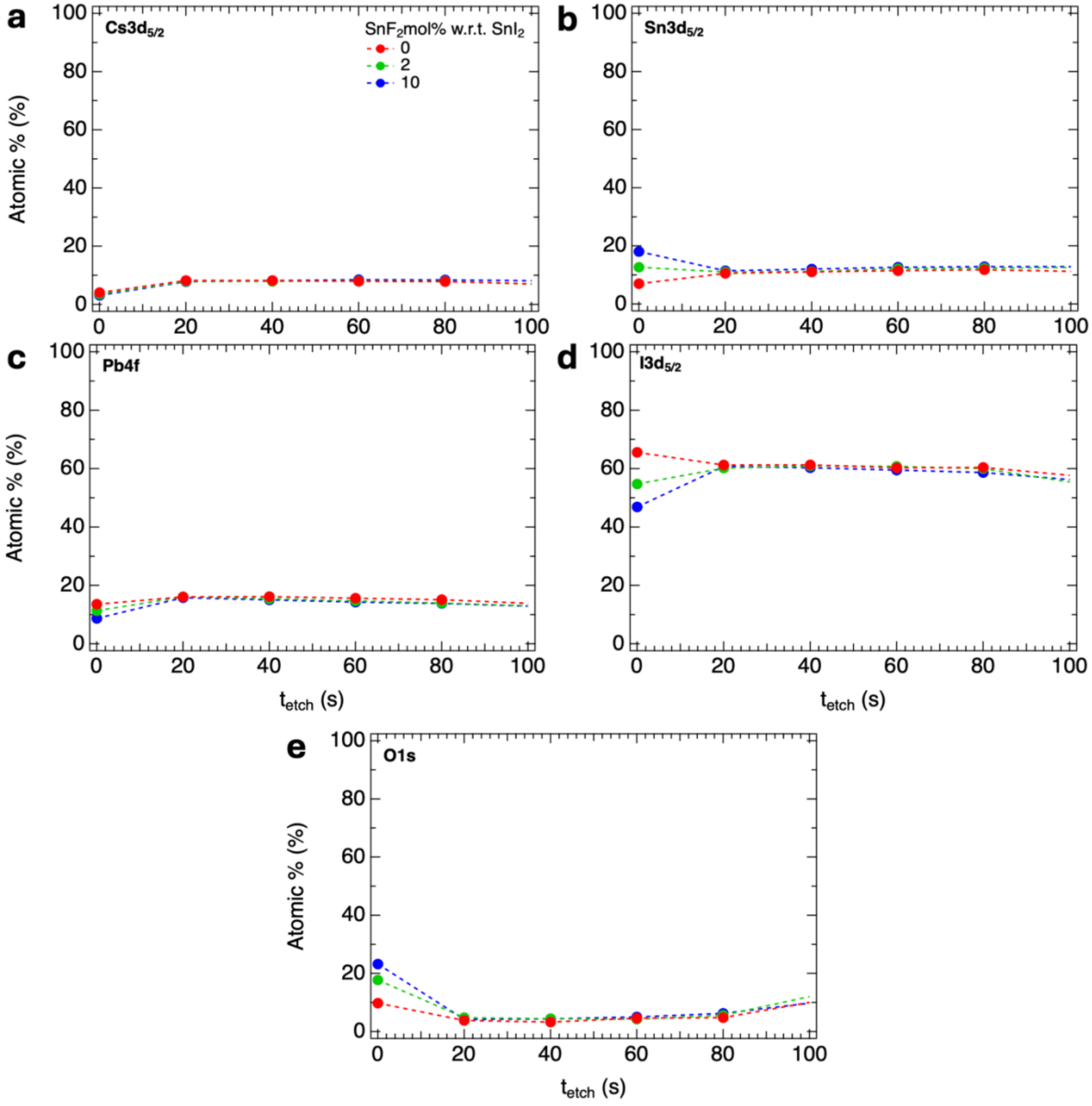

Figure S17: XPS depth analysis showing the atomic % of (a) Cs, (b) Sn, (c) Pb, (d) I and (e) O elements in $Cs_{0.25}FA_{0.75}Sn_{0.5}Pb_{0.5}I_3$ perovskite thin films with 0 (in red), 2 (in green) and 10 (in blue) mol% SnF_2 additions. All samples belong to the best-performing deposition in **Figure 3a** in the main text, produced with 0 days aged SnI_2 precursor. The XPS depth profiling is shown up to t_{etch} = 100 s, which corresponds to tens of nm from the top surface of the film.

Table S6: Elements (Sn, Pb, I) atomic % ratios calculated from XPS depth analysis in **Figure S17**, focusing on the surface (t_{etch} = 0 s) of $Cs_{0.25}FA_{0.75}Sn_{0.5}Pb_{0.5}I_3$ perovskite thin films with 0, 2 and 10 mol% SnF_2 additions.

XPS (surface)			
**SnF_2 mol%**	**0**	**2**	**10**
Sn:Pb	0.3:0.7	0.5:0.5	0.7:0.3
(Sn+Pb):I	1:3.2	1:2.3	1:1.7

Scanning Electron Microscopy (SEM) – Microstructure

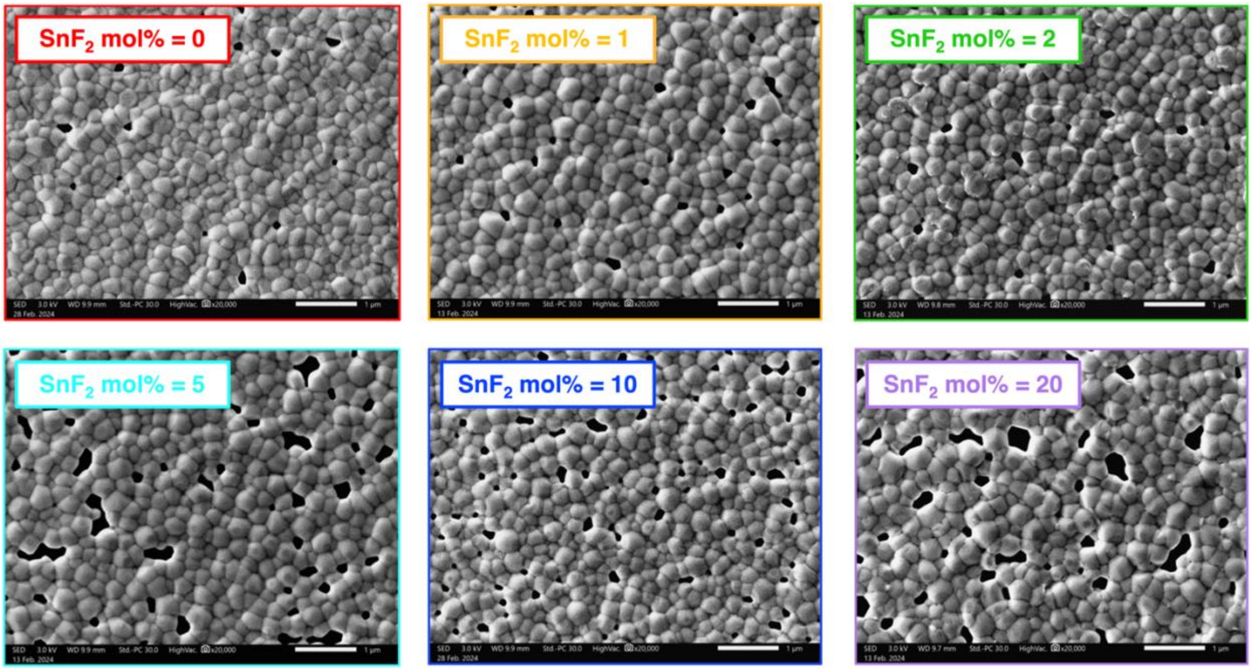

Figure S18: Secondary electrons SEM images showing the top surface topography and microstructure of $Cs_{0.25}FA_{0.75}Sn_{0.5}Pb_{0.5}I_3$ perovskite thin films with varying SnF_2 concentration. All samples belong to the best-performing deposition in **Figure 3a** in the main text, produced with 0 days aged SnI_2 precursor.

References

(1) Majumdar, S., & Devi, P. S. Synthesis of SnO_2 Nanoparticles Using Ultrasonication. *AIP Conf. Proc*. **2010***, 1276*, 1-7. **2010**. DOI: 10.1063/1.3504298

(2) Hutter, E. M. Revealing the Fate of Photo-Generated Charges in Metal Halide Perovskites, Ph.D. Dissertation, Delft University of Technology, Delft, NL, **2018**. DOI: 10.4233/uuid:f8e21539-bd26-4694-b170-6d0641e4c31a

(3) Caselli, V. M. Revealing Loss and Degradation Mechanisms in Metal Halide Perovskite Solar Cells:The Role of Defects and Trap States, Ph.D. Dissertation, Delft University of Technology, Delft, NL, **2022**. DOI: 10.4233/uuid:f8361576-f35d-4334-8bee-68a48ed70037

(4) Savenije, T. J., Guo, D., Caselli, V. M., & Hutter, E. M. Quantifying Charge-Carrier Mobilities and Recombination Rates in Metal Halide Perovskites from Time-Resolved Microwave Photoconductivity Measurements. *Adv. Energy Mater.* **2020**, *10*, 1903788. DOI: 10.1002/aenm.201903788

(5) Koning, S. J. Computational Modelling of a Resonant Microwave Cavity:A new method for obtaining sensitivity factors allowing the quantitative analysis of Time Resolved Microwave Conductivity data, M.Sc. Thesis, Delft University of Technology, Delft, NL, **2023**.

(6) Koopmans, M., Corre, V., & Koster, L. SIMsalabim: An open-source drift-diffusion simulator for semiconductor devices. *J. Open Source Softw.* **2022**, *7*, 3727. DOI: 10.21105/joss.03727

(7) Savill, K. J., Ulatowski, A. M., & Herz, L. M. Optoelectronic Properties of Tin–Lead Halide Perovskites. *ACS Energy Lett.* **2021**, *6*, 2413–2426. DOI: 10.1021/acsenergylett.1c00776

(8) Savill, K. J., Ulatowski, A. M., Farrar, M. D., Johnston, M. B., Snaith, H. J., & Herz, L. M. Impact of Tin Fluoride Additive on the Properties of Mixed Tin-Lead Iodide Perovskite Semiconductors. *Adv. Funct. Mater.* **2020**, *30,* 2005594. DOI: 10.1002/adfm.202005594

(9) Treglia, A., Ambrosio, F., Martani, S., Folpini, G., Barker, A. J., Albaqami, M. D., De Angelis, F., Poli, I., & Petrozza, A. Effect of electronic doping and traps on carrier dynamics in tin halide perovskites. *Mater. Horiz***. 2022**, *9*, 1763–1773. DOI: 10.1039/d2mh00008c

(10) Prasanna, R., Gold-Parker, A., Leijtens, T., Conings, B., Babayigit, A., Boyen, H., Toney, M. F., & McGehee, M. D. Band Gap Tuning via Lattice Contraction and Octahedral Tilting in Perovskite Materials for Photovoltaics. *J. Am. Chem. Soc.* **2017**, *139*, 11117–11124. DOI: 10.1021/jacs.7b04981

(11) Treglia, A., Prato, M., Wu, C. J., Wong, E. L., Poli, I., & Petrozza, A. Understanding the Surface Chemistry of Tin Halide Perovskites. *Adv. Funct. Mater.* **2024**, *34*, 2406954. DOI: 10.1002/adfm.202406954

(12) Wieczorek, A., Lai, H., Pious, J., Fu, F., & Siol, S. Resolving Oxidation States and X–site Composition of Sn Perovskites through Auger Parameter Analysis in XPS. *Adva. Mater. Interfaces* **2022**, *10*, 2201828. DOI: 10.1002/admi.202201828

(13) *Silicon* | *Periodic Table* | *Thermo Fisher Scientific - US*. (n.d.). https://www.thermofisher.com/nl/en/home/materials-science/learning-center/periodic-table/metalloid/silicon.html
